# Astronomia ex machina: a history, primer and outlook on neural networks in astronomy

**DOI:** 10.1098/rsos.221454

**Published:** 2023-05-31

**Authors:** Michael J. Smith, James E. Geach

**Affiliations:** Department of Physics, Astronomy and Mathematics, School of Physics, Engineering and Computer Science, University of Hertfordshire, Hatfield AL10 9AB, UK

**Keywords:** neural networks, astrophysics, machine learning

## Abstract

In this review, we explore the historical development and future prospects of artificial intelligence (AI) and deep learning in astronomy. We trace the evolution of connectionism in astronomy through its three waves, from the early use of multilayer perceptrons, to the rise of convolutional and recurrent neural networks, and finally to the current era of unsupervised and generative deep learning methods. With the exponential growth of astronomical data, deep learning techniques offer an unprecedented opportunity to uncover valuable insights and tackle previously intractable problems. As we enter the anticipated fourth wave of astronomical connectionism, we argue for the adoption of GPT-like foundation models fine-tuned for astronomical applications. Such models could harness the wealth of high-quality, multimodal astronomical data to serve state-of-the-art downstream tasks. To keep pace with advancements driven by Big Tech, we propose a collaborative, open-source approach within the astronomy community to develop and maintain these foundation models, fostering a symbiotic relationship between AI and astronomy that capitalizes on the unique strengths of both fields.

## 1. Introduction

The concept of artificial intelligence (AI) can be traced back at least 350 years to Leibniz’s *Dissertation on the Art of Combinations* [1]. Inspired by Descartes and Llull, Leibniz posited that, through the development of a ‘universal language’, all ideas could be represented by the combination of a small set of fundamental concepts, and that *new* concepts could be generated in a logical fashion, potentially by some computing machine. Leibniz’s ambitious vision ('let us calculate') has not yet been realized, but the quest to emulate human reasoning, or at least to build a machine to mimic the computational and data processing capabilities of the human brain, has persisted to this day.

It might be fair to say that the roots of AI stretch even as far back as Llull’s medieval philosophy that inspired Leibniz [2,3]. However, if we now consider AI to be a bona fide scientific discipline, then that discipline clearly emerged in the post-war years of the twentieth century, following Turing’s simple enquiry ‘can machines think?’ [4]. Somewhat philosophical in nature, Turing’s 1950 question succinctly articulates the ambition of AI, but from a nuts and bolts standpoint it took a further 5 years from Turing’s query for what one might call the first AI program—the so-called ‘Logic Theorist’—to be developed by Allen Newell, Cliff Shaw and Herbert Simon. Funded by the Research and Development (RAND) Corporation, the Logic Theorist was designed, in part, to emulate the role of a human mathematician, in that it could automate the proof of mathematical theorems. This was a breakthrough in computer science and the Logic Theorist was presented at the seminal Dartmouth Summer Research Project on Artificial Intelligence (DSRPAI) conference in 1956, now regarded as the true birth of AI as a field. Indeed, it was DSRPAI organizer John McCarthy who is credited with coining the term ‘artificial intelligence’ [5].

Natural intrigue—and clearly a good deal of fear—of the idea of AI has inspired popular culture no end, from Dick’s *Do Androids Dream Of Electric Sheep?* to Crichton’s *Westworld*, *Terminator’s ‘Skynet’* and beyond. Iain M. Banks’s Galactic civilization known as ‘The Culture’ imagines a society run by powerful ‘Minds’ whose intelligence and wisdom far exceeds that of humans, and where biological beings and machines of equivalent sentience generally coexist peacefully, cooperatively and equitably. Science fiction notwithstanding, if these dreams are even possible, we are still years away from a machine that can genuinely think for itself [6,7]. Nevertheless, the question of how one mathematically (and algorithmically) models the workings and inter-relationships of biological neurons—neural networks—and the subsequent exploration of how they can find utility as tools in the data analyst’s workshop is really what is being referred to when most people use the term ‘AI’ today.^1^ While we must always be wary of hype and buzzwordism, it is the *application* of neural networks—and the possibility of tackling hitherto intractable problems—that offers genuine reason for excitement across many disparate fields of enquiry, including astronomy.

**Figure 1.**
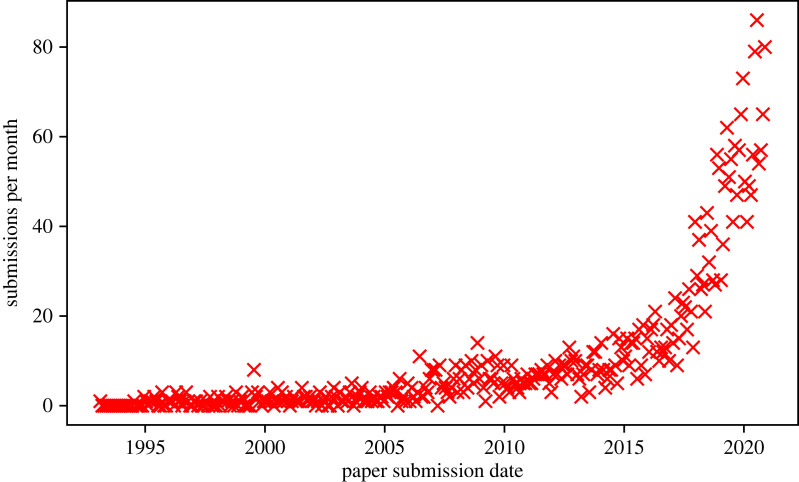
Here we see the number of arXiv:astro-ph submissions per month that have abstracts or titles containing one or more of the strings: ‘machine learning’, ‘ML’, ‘artificial intelligence’, ‘AI’, ‘deep learning’ or ‘neural network’. The raw data are in the public domain and are available at https://www.kaggle.com/Cornell-University/arxiv.

Astronomers have made use of artificial neural networks (ANNs) for over three decades. In 1994, Ofer Lahav, an early trailblazer, wryly identified the ‘neuro-skeptics’—those resistant to the use of such techniques in serious astrophysics research—and argued that ANNs ‘should be viewed as a general statistical framework, rather than as an estoteric approach’ [8]. Unfortunately, this scepticism has persisted. This is despite the recent upsurge in the use of neural networks (and machine learning in general) in the field, as illustrated in figure 1. This scepticism also stands contrary to achievements within astronomy that would not be possible without the use of ANNs, such as photometric redshift estimation (e.g. [9,10]), astronomical object identification and clustering at scale (e.g. [11]) and entirely data-driven simulation (e.g. [12,13]). Most of the criticism of machine learning techniques, and deep learning^2^ in particular, is levelled at the perceived ‘black box’ nature of the methodology. In this review, we provide a primer on how deep neural networks are constructed, and the mathematical rules governing their learning, which we hope will serve as a useful resource for neuro-sceptics. Nevertheless, we must recognize that a unified theoretical picture of how deep neural networks work does not yet exist. This remains a point of debate even within the deep learning community. For example, Yann LeCun responding to Ali Rahimi’s ‘Test of Time’ award talk at the 31st Conference on Neural Information Processing Systems (NIPS) remarked:

Ali gave an entertaining and well-delivered talk. But I fundamentally disagree with the message. The main message was, in essence, that the current practice in machine learning is akin to ‘alchemy’ (his word). It’s insulting, yes. But never mind that: It’s wrong! Ali complained about the lack of (theoretical) understanding of many methods that are currently used in ML, particularly in deep learning ... Sticking to a set of methods just because you can do theory about it, while ignoring a set of methods that empirically work better just because you don’t (yet) understand them theoretically is akin to looking for your lost car keys under the street light knowing you lost them someplace else. Yes, we need better understanding of our methods. But the correct attitude is to attempt to fix the situation, not to insult a whole community for not having succeeded in fixing it yet. This is like criticizing James Watt for not being Carnot or Helmholtz [14].

Philosophical concerns aside, LeCun’s fundamental point is that deep learning ‘works’ and therefore we should use it, even if we do not fully understand it. If one were being uncharitable, we could make similar arguments about the ΛCDM paradigm.

**Figure 2.**
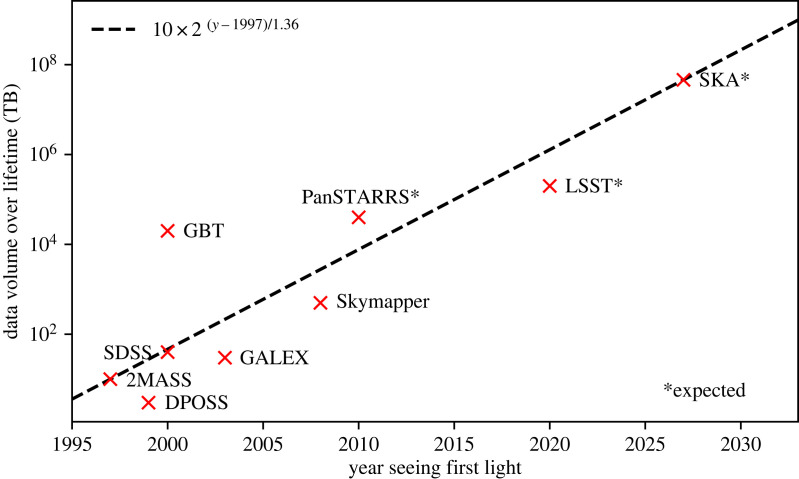
The data volume output of a selection of astronomical surveys over their lifetimes. We can see the astronomical survey data volume doubles every 16 months. Data are taken from Zhang & Zhao [19].

It is clear that in every field that deep learning has infiltrated we have seen a reduction in the use of specialist knowledge, to be replaced with knowledge automatically derived from data. We have already seen this process play out in many ‘applied deep learning’ fields such as computer Go [15], protein folding [16], natural language processing [17] and computer vision [18]. We argue that astronomy’s data abundance corrals it onto a path no different to that trodden by other applied deep learning fields. This abundance is not a passing phase; the total astronomical data volume is already large and will increase exponentially in the coming years. We illustrate this in figure 2, where we present a selection of astronomical surveys and their estimated data volume output over their lifetimes [19]. And this is not even considering data associated with ever larger and more detailed numerical simulations (e.g. [20–22]). The current scale of the data volume already poses an issue for astronomy as many classical methods rely on human supervision and specialist expertise, and the increasing data volume will make exploring and exploiting these surveys through traditional human supervised and semi-supervised means an intractable problem. Of serious concern is the possibility that we will miss—or substantially delay—interesting and important discoveries simply due to our inability to accurately and consistently interrogate astronomical data at scale. Deep learning has shown great promise in automating information extraction in various data-intensive fields, and so is ideally poised as a solution to the challenge of processing ultra-large-scale astronomical data. But we do not need to stop there. This review’s outlook ventures a step further, and argues that astronomy’s wealth of data should be considered a unique opportunity, and not merely an albatross.

Since astronomical connectionism’s^3^ humble beginnings in the late 1980s, there have been numerous excellent reviews on the application of artificial neural networks to astronomy (e.g. [23–25]). We take an alternative approach to previous literature reviews and survey the field holistically, in an attempt to paint astronomical connectionism’s ‘Big Picture’ with broad strokes. While we cannot possibly include all works within astronomical connectionism,^4^ we hope that this review serves as a historical background on astronomy’s ‘three waves’ of increasingly automated connectionism, as well as presenting a general primer on neural networks that may assist those seeking to explore this fascinating topic for the first time.

In §§2 and 3, we explore initial work on multi-layer perceptrons within astronomy, where models required manually selected emergent properties as input. In §§4 and 5, we explore the second wave, which coincided with the dissemination of convolutional neural networks and recurrent neural networks—models where the multi-layer perceptron’s manually selected inputs are replaced with raw data ingestion. In the third wave that is happening now we are seeing the removal of human supervision altogether with deep learning methods inferring labels and knowledge directly from the data, and we explore this wave in §§6–8. Finally, in §9, we look to the future and predict that we will soon enter a fourth wave of astronomical connectionism. We argue that if astronomy follows the pattern of other applied deep learning fields we will see the removal of expertly crafted deep learning models, to be replaced with fine-tuned versions of an all-encompassing ‘foundation’ model. As part of this fourth wave, we argue for a symbiosis between astronomy and connectionism, a symbiosis predicated on astronomy’s relative data wealth and deep learning’s insatiable data appetite. Many ultra-large datasets in machine learning are proprietary or of poor quality, and so there is an opportunity for astronomers as a community to develop and provide a high-quality multi-modal public dataset. In turn, this dataset could be used to train an astronomical foundation model to serve state-of-the-art downstream tasks. Owing to foundation models’ hunger for data and compute, a single astronomical research group could not bring about such a model alone. Therefore, we conclude that astronomy as a discipline has slim chance of keeping up with a research pace set by the Big Tech goliaths—that is, unless we follow the examples of EleutherAI and HuggingFace and pool our resources in a grassroots open-source fashion.

Before moving on, we must first admit to our readers that we have not been entirely honest with them. The abstract of this review has not been written by us. It was generated by prompting OpenAI’s generative pretrained transformer 4 (‘GPT-4’) neural network-based foundation model with this paper’s introduction [26,27]. To be precise, we prompted the GPT-4 engine provided by ‘ChatGPT Plus’ with all the text in §1 up until this paragraph in raw LaTeX format. We then appended the following prompt to the introduction text:

Write an abstract for the above text that will catch the reader’s eye, and make them interested in the paper. Make the abstract 160 words or less, and touch on the value of GPT-like models in astronomy.

We did not alter the GPT-generated output whatsoever. We explore these foundation models and their possible astronomical uses in more detail in §9.

## 2. A primer on artificial neurons

In 1943 McCulloch & Pitts [28] proposed the first computational model of a biological neuron (MP neuron; [28]). Their model consisted of a set of binary inputs *x*_*i*_ ∈ {0, 1} and a single binary output *y* ∈ {0, 1}. Their model also defines a single ‘inhibitory’ input I∈{0,1} that blocks output if I=1. If the sum of the inputs exceeds a threshold value Θ, the MP neuron ‘fires’ and outputs *y* = 1. Mathematically, we can write the MP neuron function as

$$\text{MP}(x)=\left\{ \begin{array}{cc} \text{1}\; & \text{if}\;\sum_{i=1}^{n}x_{i}>\Theta \text{ and }I=0,\;\\ \text{0}\; & \text{otherwise.}\; \end{array}\right.$$

The MP neuron is quite a powerful abstraction. Single MP neurons can calculate simple Boolean functions, and more complicated functions can be calculated when many MP neurons are chained together. However, there is one show-stopping issue: the MP neuron is missing the capacity to learn. Rosenblatt [29] addressed this by combining the MP neuron with Hebb’s neuronal wiring theory^5^ [30], and we will explore a related training formulation in the next subsection.

### 2.1. The perceptron

This subsection aims to provide the reader a foundation and intuition for the gradient-based learning that dominates contemporary neural network architectures. Therefore, we diverge from Rosenblatt’s original learning algorithm and instead describe a gradient-based training algorithm. The interested reader will find an analysis of Rosenblatt’s original learning algorithms in the ‘Mathematical analysis of learning in the perceptron’ section of Rosenblatt [29].

**Figure 3.**
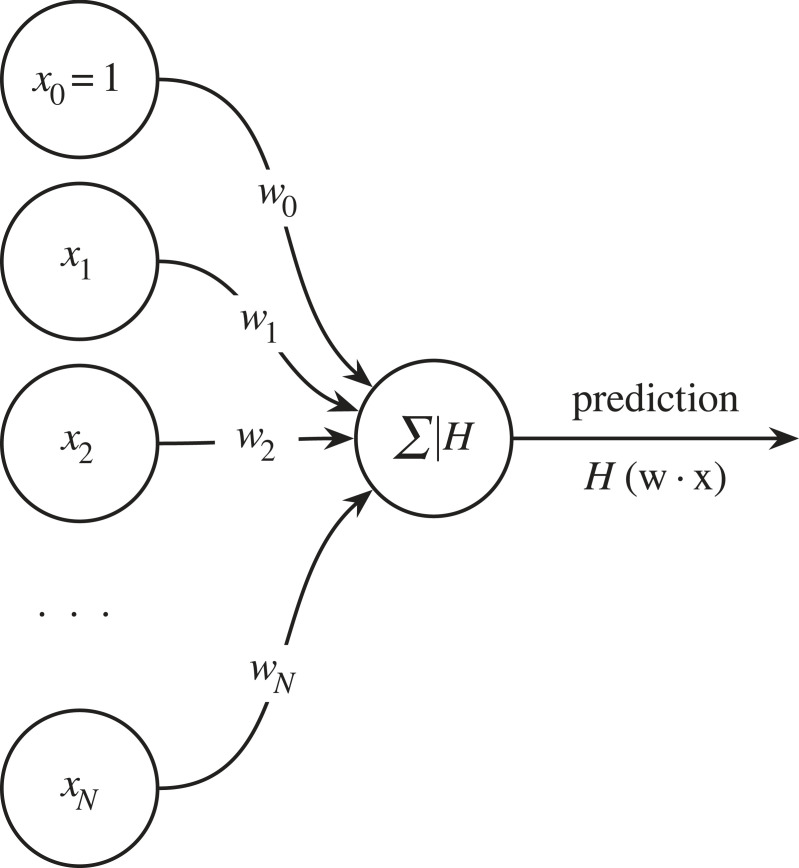
A single neuron (or perceptron) with a bias *w*_0_, inputs *x*_1_, *x*_2_, …, *x*_*N*_, and weights *w*_1_, *w*_2_, …, *w*_*N*_.

Like the MP neuron, the perceptron takes a number of numeric inputs (*x*_*i*_). However, unlike the MP neuron, each one of these inputs is multiplied by a corresponding weight (*w*_*i*_) signifying the importance the perceptron assigns to a given input. As shown in figure 3, we can then sum this list of products and pass it into an ‘activation function’. Let us use the Heaviside step function as our activation function,

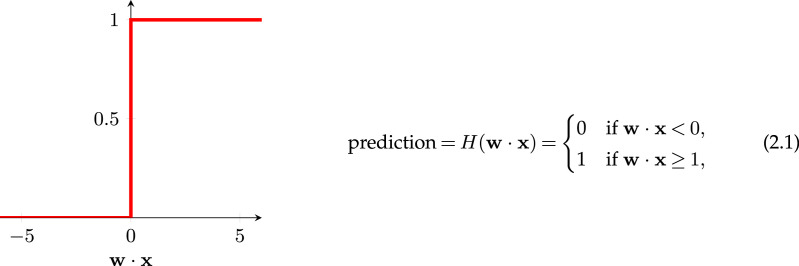
where **x** is a set of inputs, and **w** is a set of ‘weights’ that represent the importance of each input.

To concretize how we could train our perceptron, we will use an example. Let us say that we want to automatically label a set of galaxy images as either ‘spiral’ or ‘elliptical’. To do this, we first need to compile a training dataset of galaxy images. This training set would consist of spiral and elliptical galaxies, and each image would have a ground truth label *y*—say ‘0’ for a spiral galaxy and ‘1’ for an elliptical. To train our perceptron, we randomly choose one image from the training set, and feed it to the perceptron, with the numerical value of each pixel corresponding to an input {*x*_1_, …, *x*_*N*_}. These inputs are multiplied by their corresponding weight {*w*_1_, …, *w*_*N*_}. A bias term (*b* = *w*_0_ *x*_0_, where *x*_0_ = 1) is also added to the inputs, which allows the neuron to shift its activation function linearly. Since we do not want our perceptron to have any prior knowledge of the task, we initialize the weights at random. The resulting products are then summed. Finally, our activation function *H* transforms **w** · **x** and produces a prediction *p*. We then compare *p* with *y* via a ‘loss function,’ which is a function that measures the difference between *p* and *y*. The loss can be any differentiable function, so for illustration purposes we will define it here as the L1 loss: L(y,p)=|y−p|. Now that we can compare with the ground truth, we need to work out how a change in one of our weights affects the loss (that is, we want to find ∂L/∂w). We can calculate this change with the chain rule

2.2 $$\frac{\partial L}{\partial w}=\frac{\partial L}{\partial p}\frac{\partial p}{\partial w},$$

and since *p* = *H*(**w** · **x**) and ∂*p*/∂**w** = *H*′**x**^*T*^ we get

$$\frac{\partial L}{\partial w}=\frac{\partial L}{\partial p}\;⊙(H^{\prime }x^{T}),$$

where ⊙ is the distributive Hadamard product. Thus, we can update the weights to decrease the loss function,

$$\begin{array}{rl} w_{\text{next}} & =w-\eta \frac{\partial L}{\partial w}\\ & =w-\eta \frac{\partial L}{\partial p}⊙(H^{\prime }x^{T}), \end{array}$$

where *η* is the learning rate.^6^ If we repeat this process our perceptron will get better and better at classifying our galaxies!

While we provide the above example for illustrative purposes, we will need a more powerful algorithm to produce a useful classifier of galaxy morphology. This need is perhaps most famously discussed in *Perceptrons: An Introduction to Computational Geometry* ([31], e.g. §13.0). Minsky & Papert show that the single-layer perceptron is only able to calculate linearly separable functions, among other limitations. Their book (alongside a consensus that AI had failed to deliver on its early grandiose promises) delivered a big blow to the connectionist school of artificial intelligence.^7^ In the years following Minsky & Papert [31], governmental and industry funding was pulled from connectionist research laboratories, ushering in the first ‘AI winter’.^8^

**Figure 4.**
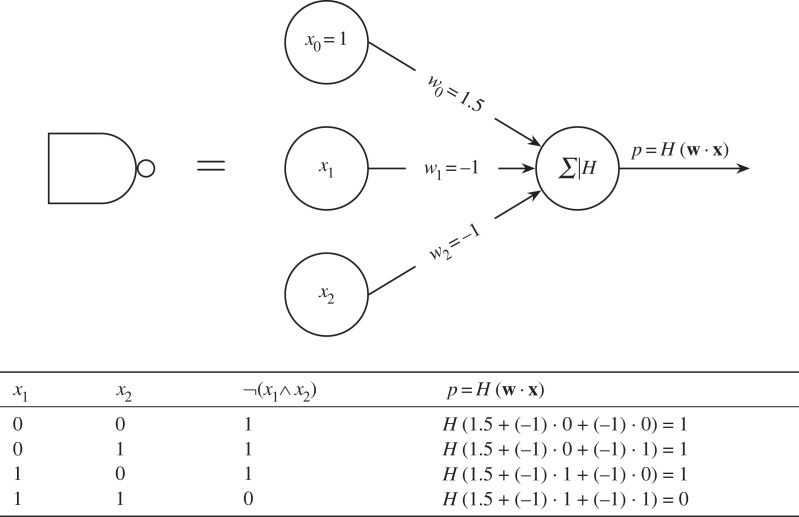
If we define *H*(**w** · **x**) as in equation (2.1), we can set a perceptron’s weights so that it is equivalent to the NAND gate.

Yet, as exemplified in Rosenblatt ([36], §5.2, theorem 1) it was known at the time that multi-layer perceptrons could calculate nonlinearly separable functions (such as the ‘exclusive or’). We can prove intuitively that a set of neurons can calculate *any* function: a perceptron can perfectly emulate a NAND gate (figure 4), and the singleton set {NAND} is functionally complete. Since we can combine a set of NAND gates to calculate any function, *we must also be able to combine a set of neurons to calculate any function*. This result is also explored in a more formal proof by both Cybenko [37] and Hornik *et al.* [38]. They show that an infinitely wide neural network can calculate any function. Similarly, Lu *et al.* [39] show that an infinitely deep neural network is a universal approximator. Such a group of neurons is known as the multi-layer perceptron (MLP). Unfortunately, we cannot simply stack perceptrons together as we are missing one vital ingredient: a way to train the network! At the time of Minsky & Papert’s treatise on perceptrons, there was no widely known algorithm (in the West; see [34]) that could train such a multi-layer network. In Minsky & Papert’s own words:

Nevertheless, we consider it to be an important research problem to elucidate (or reject) our intuitive judgment that the extension [from one layer to many] is sterile. Perhaps some powerful convergence theorem will be discovered, or some profound reason for the failure to produce an interesting ‘learning theorem’ for the multilayered machine will be found. (Minsky & Papert [31], §13.2 on MLPs)

The field had to wait almost two decades for such an algorithm to become widespread. In the next subsection, we will explore backpropagation, the algorithm that ultimately proved Minsky and Papert’s intuition wrong.

### 2.2. The multi-layer perceptron

**Figure 5.**
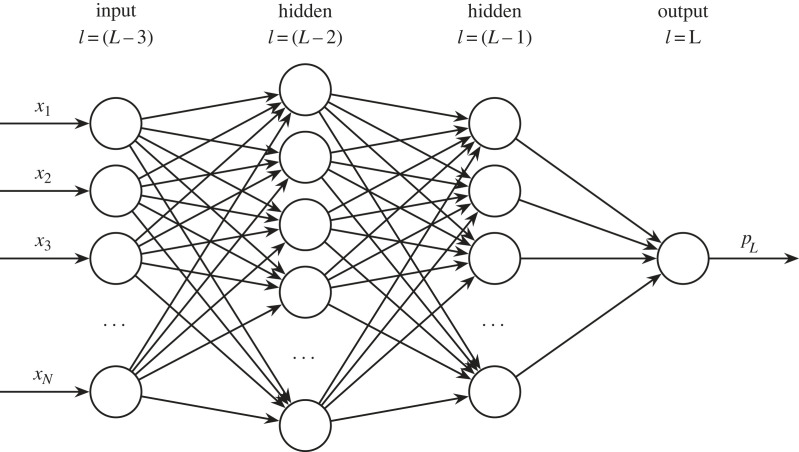
The multi-layer perceptron, or artificial neural network. The depicted network has two hidden layers. It takes *N* inputs *x*_1_, *x*_2_, …, *x*_*N*_, and outputs a prediction *p*_*L*_. Note that here we omit the explicit bias terms (i.e. *w*_0_).

Grouping many artificial neurons together may result in something resembling figure 5. This network consists of an input layer, two intermediate ‘hidden’ layers, and an output layer. As in the previous section, let us say that we want a classifier that can classify a set of galaxy images into elliptical and spiral types. In an MLP similar to figure 5, a neuron would be assigned to each pixel in a galaxy image. Each neuron would take the numeric value of that pixel, and propagate that signal forward into the network. The next layer of neurons does the same, with the input being the previous layer’s output. This process continues until we reach the output layer. In a binary classification task like our galaxy classifier, this layer outputs a value between zero and one. Thus, if we define a spiral galaxy as zero, and an elliptical galaxy as one, we would want the network output to be near zero for a spiral galaxy input (and vice versa).

**Figure 6.**
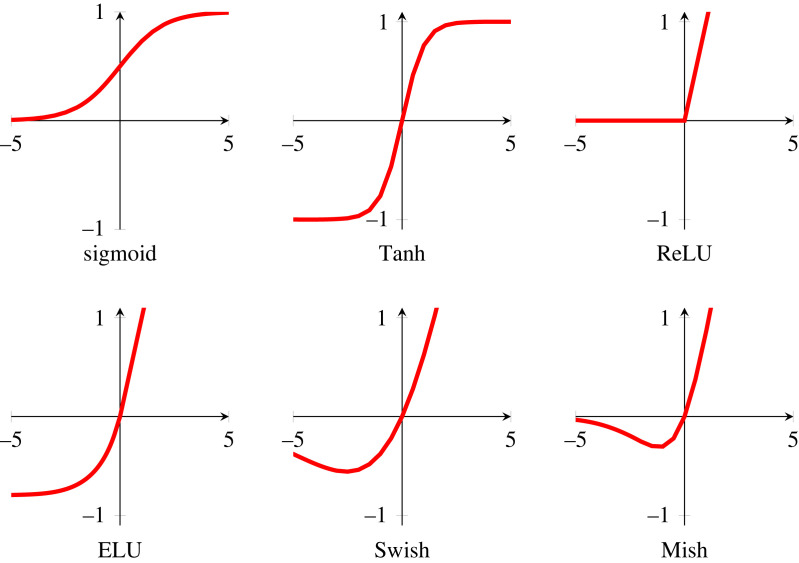
A curated selection of activation functions. In all plots, the *x*-axis is the input, and the y-axis is the output. The rectified linear unit (ReLU) activation function was first introduced in the context of neural networks in Fukushima [46] and later rediscovered, named and popularized in Nair & Hinton [47]. The exponential linear unit (ELU), Swish and Mish activations were, respectively, introduced in Clevert *et al.* [48], Ramachandran *et al.* [49] and Misra [50].

In §2.1, we found the change we needed to apply to a single neuron’s weights to make it learn from a training example. We can train an MLP in a similar way by employing the reverse mode of automatic differentiation (or backpropagation) to learn from our galaxy training dataset [40–42].^9^ We want our network to learn when it makes both a correct and incorrect prediction, so we define our activation function as a smoothed version of the Heaviside step function. This ensures that a signal is present in the derivative no matter which values are input. This activation function is known as the ‘sigmoid’ function, and is shown in figure 6. As in §2.1, we define a loss function L(y,p) that describes the similarity between a ground truth (*y*) and a prediction (*p*). We also define a neuron’s activation function as φ(**w** · **x**) where **w** · **x** is the weighted sum of a neuron’s inputs. Following from equation (2.2)

$$\frac{\partial L}{\partial w_{l}}=\frac{\partial L}{\partial p_{l}}\frac{\partial p_{l}}{\partial w_{l}},$$

where *l* is a layer in the MLP. In the same way as in §2.1, we can calculate an MLP’s final layer’s (*l* = *L*) weight updates in terms of known values

2.3 $$\frac{\partial L}{\partial w_{L}}=\frac{\partial L}{\partial p_{L}}⊙(\varphi_{L}^{\prime }p_{L-1}^{T}),$$

where **p**_*L*−1_ are the outputs from the previous layer. To calculate the (*L* − 1)th layer’s weight updates, we use the chain rule

$$\frac{\partial L}{\partial w_{L-1}}=\frac{\partial L}{\partial p_{L}}\frac{\partial p_{L}}{\partial p_{L-1}}\frac{\partial p_{L-1}}{\partial w_{L-1}}.$$

Likewise for the (*L* − *n*)th layer

$$\frac{\partial L}{\partial w_{L-n}}=\frac{\partial L}{\partial p_{L}}\left(\prod_{i=1}^{n}\frac{\partial p_{L+1-i}}{\partial p_{L-i}}\right)\frac{\partial p_{L-n}}{\partial w_{L-n}}.$$

Now we can start plugging in some known values. Since **p**_*l*_ = φ_*l*_(**w**_*l*_ · **p**_*l*−1_), it follows that $\partial p_{l}/\partial p_{l-1}=\varphi_{l}^{\prime }w_{l}^{T}$, and $\partial p_{l}/\partial w_{l}=\varphi_{l}^{\prime }p_{l-1}^{T}$. So

2.4 $$\frac{\partial L}{\partial w_{L-n}}=\frac{\partial L}{\partial p_{L}}⊙\left(\prod_{i=1}^{n}\varphi_{L-i}^{\prime }w_{L-i}^{T}\right)\;(\varphi_{L-n}^{\prime }p_{L-n-1}^{T}).$$

Combining equation (2.3) with equation (2.4) we get the weight update algorithm for the (*L* − *n*)th layer of the MLP

2.5 $$w_{\text{next}}=w-\eta \left\{ \begin{array}{cc} \frac{\partial L}{\partial p_{L}}⊙(\varphi_{L}^{\prime }p_{L-1}^{T}),\; & \text{for }n=0,\;\\ \frac{\partial L}{\partial p_{L}}⊙\left(\prod_{i=1}^{n}\varphi_{L-i}^{\prime }w_{L-i}^{T}\right)\;(\varphi_{L-n}^{\prime }p_{L-n-1}^{T}),\; & \text{for }n>0.\; \end{array}\right.$$

With this equation^10^ in hand, we can use the same technique described earlier in this section and in §2.1 to update the network’s weights with each galaxy image to decrease the loss function L. Again, as L is minimized, our MLP will classify our elliptical and spiral galaxy images with increasing accuracy.

## 3. Astronomy’s first wave of connectionism

Connectionism was first discussed within astronomy in the late 1980s, after the popularization of backpropagation (see footnote 9) and the consequent passing of the first ‘AI winter’. Two radical studies emerged in 1988 that recognized areas where astronomy could benefit from the use of ANNs [51,52]. Together, they identified that astronomical object classification,^11^ and telescope scheduling could be solved through the use of an ANN. These studies were followed by a rapid broadening of the field, and the application of connectionism to many disparate astronomical use cases ([23] and references therein). In this section, we will outline areas where MLPs found an early use in astronomy.

### 3.1. Classification problems

Odewahn *et al.* [53] classified astronomical objects into star and galaxy types. These were taken from the Palomar Sky Survey Automated Plate Scanner catalogue [54]. To compile their dataset, they first extracted a set of emergent image parameters from the scanned observations. These parameters included the diameter, ellipticity, area and plate transmission. The parameters were then used to train both a linear perceptron and a feedforward MLP to classify the objects into stars or galaxies. Odewahn *et al.* [53] found that their best performing model could classify galaxies with a completeness of 95% for objects down to a magnitude less than 19.5. This work was followed by many more studies on the star/galaxy classification problem (e.g. [55–58]). Galaxy morphological type classification was explored in the early 1990s. Storrie-Lombardi & Lahav [59] describe an MLP that takes as input a selected set of 13 galaxy summary statistics, and uses this information to classify a galaxy into one of five morphological types. Storrie-Lombardi & Lahav [59] report a top one accuracy of 64%, and a top two accuracy of 90%. This pilot study was followed by several studies from the same group that confirmed that MLPs are effective automatic galaxy morphological classifiers ([60–65], see §5 for a continuation of this line of research).

MLPs were also used in other classification tasks; here we highlight a few further areas where MLPs were applied. Von Hippel *et al.* [66] classified stellar spectra into temperature types, and Klusch & Napiwotzki [67] did the same for Morgan–Keenan system types. Chon [68] described the use of an MLP to search for and classify muon events (and therefore neutrino observations) in the Sudbury Neutrino Observatory. Quasar classification has been explored in several studies [69–71]. Seminally, Carballo *et al.* [69] used an MLP to select quasar candidates given their radio flux, integrated-to-peak flux ratio, photometry and point spread function in the red and blue bands, and their radio-optical position separation. They found good agreement between their model and that of the decision tree described in White *et al.* [72], confirming MLPs as a competitive alternative to more traditional machine learning. As part of the Supernova Photometric Classification Challenge (SPCC, [73]), Karpenka *et al.* [74] proposed the use of a neural network to classify supernovae into Type-1a/non-Type-1a classes. To classify their light curves, they first used a hand-crafted fitting function, and then trained their MLP on the fitted coefficients. They found that their model was competitive with other, more complex models trained on the SPCC dataset. From the studies discussed in this section, we can safely conclude that MLPs are effective classifiers of astronomical data, when given important parameters extracted by an expert guide.

### 3.2. Regression problems

MLPs were also used in regression problems. Angel *et al.* [75] applied them first to adaptive telescope optics. They trained their MLP on 250 000 simulated in focus and out of focus observations of stars as seen by the Multiple Mirror Telescope (MMT). From the flattened 13 × 13 pixel observations, their network predicted the piston position and tilt required for each of the MMT’s mirrors to bring the stars into focus. After the application of these corrections, the authors were able to recover the original profile. In follow-up studies, Sandler *et al.* [76] and Lloyd-Hart *et al.* [77] proved that Angel *et al*.’s MLP worked on the real MMT.

Photometric redshift estimation was explored in many concurrent studies (e.g. [9,10,65,78,79]). Firth *et al.* [10] trained a neural network to predict the redshift of galaxies contained in the Sloan Digital Sky Survey (SDSS) early data release [80]. The galaxies were input to the neural network as a set of summary parameters, and the output was a single float representing the galaxy redshift. They found their network attained a performance comparable to classical techniques. Extending and confirming the work by Firth *et al.* [10], Ball *et al.* [65] used an MLP to predict the redshift of galaxies contained in the SDSS’s first data release [81]. They also showed that MLPs were capable of predicting the galaxies’ spectral types and morphological classifications.

Of course, MLPs have been used more widely in astronomical regression tasks. Here we will cherry pick a few studies to show the MLP’s early breadth of use. Sunspot maxima prediction was carried out by Koons & Gorney [82]. They found their MLP-based method was capable of predicting the number of sunspots when trained on previous cycles. Bailer-Jones *et al.* [83] predicted the effective temperature of a star from its spectrum. Auld *et al.* [84,85] applied MLPs to cosmology, demonstrating that MLPs are capable of predicting the cosmic microwave background power spectra and matter power spectra when given a set of cosmological parameters. Nørgaard-Nielsen & Jørgensen [86] used an MLP to remove the foreground from microwave temperature maps. From the studies discussed in this section, we can see that MLPs are effective regressors of astronomical data, when given significant parameters extracted by an expert guide.

## 4. Contemporary supervised deep learning

There are some issues with MLPs. Primarily they do not scale well to high-dimensional datasets. For example, if our dataset consists of images with 128 × 128 pixels, we will need 16 384 neurons in the MLP’s input layer alone! As we move into the hidden layers, this scaling issue only gets worse. Also, since MLPs must take an unrolled image as an input, they disregard any spatial properties of their training images, and so either need a substantial amount of training data to classify or generate large images,^12^ or an expert to extract descriptive features from the data in a preprocessing step. We can see this issue writ large in the previous section—most of the MLP applications described in §3 require an expert to extract features from the data for the network to then train on! This drawback is not ideal; what if there are features within the raw data that are not present in these cherry-picked statistics? In that case, it would be preferable to let the neural network take in the raw data as input, and then learn which features are the most descriptive. We will discuss neural network architectures that solve both the MLP scaling problem and the expert reliance problem in this section. After we have explored these architectures in general, we will discuss their application to astronomical problems in §5.

### 4.1. Convolutional neural networks

**Figure 7.**
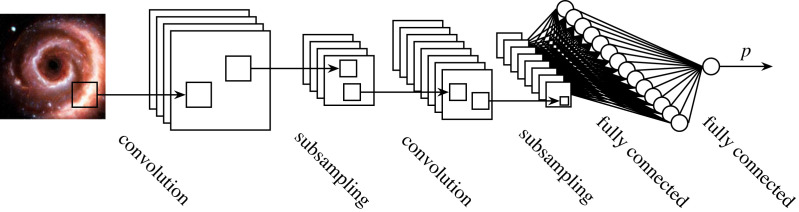
A convolutional neural network classifying a spiral galaxy image.^13^

Unlike the MLP described in the previous section, convolutional neural networks (CNNs; introduced in Fukushima [46] and first combined with backpropagation in LeCun *et al.* [93]) do not entirely consist of fully connected layers, where each neuron is connected to every neuron in the previous and subsequent layers. Instead, the CNN (such as the one depicted in figure 7) uses convolutional layers in place of the majority (or all) of the dense layers.

We can think of a convolutional layer as a set of learnt ‘feature filters’. These feature filters perform a local transform on input imagery. In classical computer vision, these filters are hand crafted, and perform a predetermined function, such as edge detection or blurring. By contrast, a CNN learns the optimal set of filters for its task (say, galaxy classification). Equation (4.1) shows two different convolution^14^ operators being performed on an array.

4.1 $$\begin{array}{cc} \left[\begin{array}{ccccc} 39 & 57 & 86 & 9 & 26\\ 90 & 74 & 63 & 87 & 98\\ 79 & 34 & 26 & 16 & 46\\ 67 & 61 & 96 & 1 & 79\\ 33 & 47 & 15 & 49 & 29 \end{array}\right]⋆\left[\begin{array}{ccc} 0 & 0 & 0\\ 0 & 0 & 0\\ 0 & 0 & 1 \end{array}\right] & =\;\left[\begin{array}{ccc} 26 & 16 & 46\\ 96 & 1 & 79\\ 15 & 49 & 29 \end{array}\right]\\ \left[\begin{array}{ccccc} 39 & 57 & 86 & 9 & 26\\ 90 & 74 & 63 & 87 & 98\\ 79 & 34 & 26 & 16 & 46\\ 67 & 61 & 96 & 1 & 79\\ 33 & 47 & 15 & 49 & 29 \end{array}\right]⋆\left[\begin{array}{ccc} 1 & 0 & 0\\ 0 & 1 & 0\\ 0 & 0 & 1 \end{array}\right] & =\;\left[\begin{array}{ccc} 139 & 136 & 219\\ 220 & 101 & 158\\ 155 & 179 & 56 \end{array}\right] \end{array}$$

In the above equation, the operation is represented as a matrix. In a CNN, the matrix is a set of neuronal weights. As shown in figure 7, there are multiple feature maps in a convolutional layer, each containing a set of weights independent to the other feature maps, and learning to extract a different feature. Owing to the convolution operator’s inbuilt translational equivarience, these features can be detected by the convolutional layer no matter where they are in the image. As in the MLP described in the previous section, the weights are updated using backpropagation to minimize a loss function. We will discuss astronomical applications of CNNs in §5, after we introduce modern CNN architectures.

### 4.2. Recurrent neural networks

**Figure 8.**
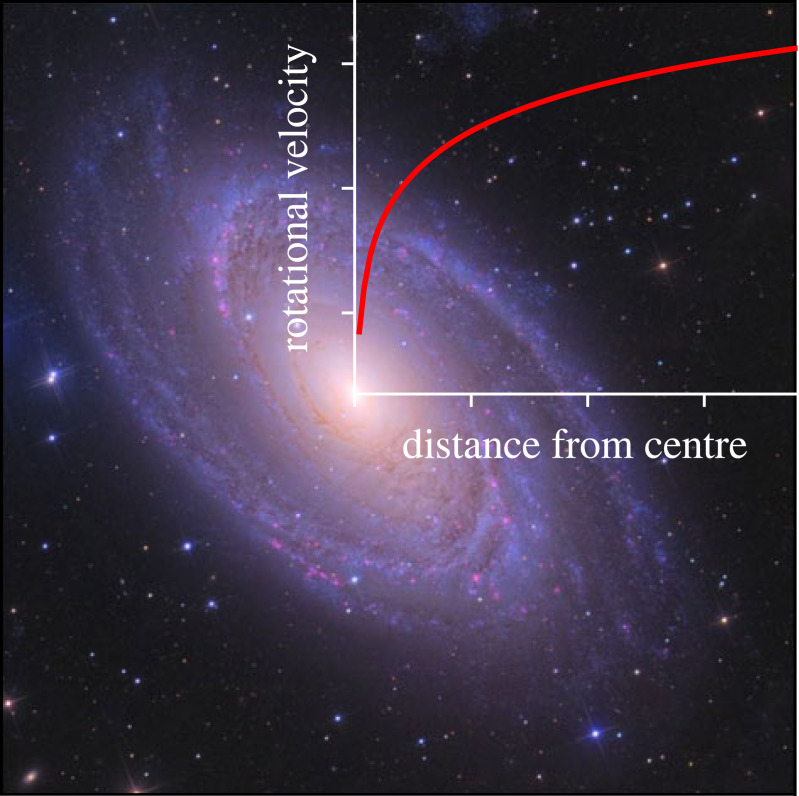
An example of a galaxy rotation curve, plotted over an image of Messier 81 [96].

Standard feedforward neural networks like the MLP (§2.2) and CNN (§4.1) generate a fixed-size vector given a fixed-size input.^15^ But, what if we want to classify or generate a variably sized vector? For example, we might want to classify a galaxy’s morphology given its rotation curve. A rotation curve describes the velocity of a galaxy’s visible stars versus their distance from the galaxy’s centre. Figure 8 shows a possible rotation curve for Messier 81. A rotation curve’s length depends on the size of its galaxy, and due to this variable length, and the fact that MLPs take a fixed-size input, we cannot easily use an MLP for classification. Recurrent neural networks (RNNs), however, can take a variable length input and produce a variable length output. An RNN differs from a feedforward MLP by having a hidden state that acts as a ‘memory’ store of previously seen information. As the RNN encounters new data, its weights are altered through the backpropagation through time algorithm (BPTT; [97] and references therein. Also see footnote 9).

**Figure 9.**
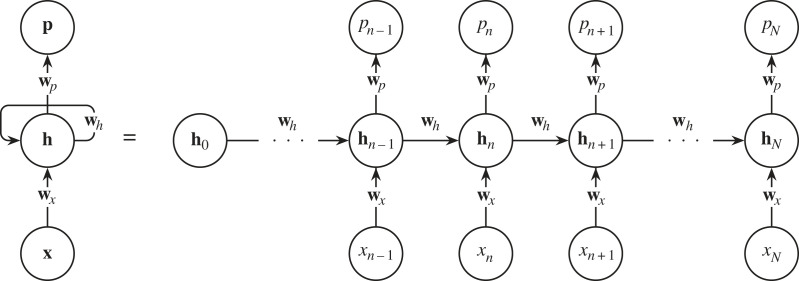
A recurrent neural network with weights {**w**_*x*_, **w**_*p*_, **w**_*h*_}, a hidden state **h**_*n*_, inputs **x** and a prediction *p*_*n*=*N*_ is unrolled into its constituent processes.

We can use an RNN similar to figure 9 to classify our rotation curves. We express the rotation curve as a list {*x*_1_, *x*_2_, …, *x*_*N*_}, with each *x* being a measurement of the rotational velocity at a certain radius. Then we feed this list into the RNN sequentially in the same way as shown in figure 9. The RNN will produce an output for each *x* fed to it, but we ignore those until we feed in *x*_*N*_, the rotational velocity furthest from the galaxy’s centre. When we feed in *x*_*N*_, the RNN produces a prediction *p*_*N*_, which we can then compare with a ground truth *y*_*N*_ via a loss function $L_{N}$. In our case, *y* is an integer label representing the galaxy’s morphological class. The comparison $L_{N}(y_{N},p_{N})$ is a function that represents the distance between the RNN prediction and the ground truth. We can then reduce $L_{N}(y_{N},p_{N})$ by updating the RNN’s weights through BPTT so that the weights {**w**_*x*_, **w**_*p*_, **w**_*h*_} follow $\nabla L_{N}$ downwards. As we do this, our RNN will improve its galaxy classifications.

BPTT’s mathematical derivation is akin to the one we explored in §2.2, and we will quickly derive it here for posterity. Let us first look at the forward propagation equations,

$$\begin{array}{rl} & L_{n}=|y_{n}-p_{n}|,\\ & p_{n}=\varphi (w_{\;p}\cdot h_{n})\\ \mathrm{and}\;\;\;\;\;\;\;\;\; & h_{n}=\phi (w_{h}\cdot h_{n-1}+w_{x}\cdot x_{n}). \end{array}$$

From these we see that we need to express $\partial L_{n}/\partial w_{\;p}$, $\partial L_{n}/\partial w_{h}$ and $\partial L_{n}/\partial w_{x}$ as known values to train the network. $\partial L_{n}/\partial w_{\;p}$ is relatively easy; via the chain rule, and the fact that $\partial p_{n}/\partial w_{\;p}=\varphi^{\prime }h_{n}^{T}$

4.2 $$\begin{array}{rl} \frac{\partial L_{n}}{\partial w_{\;p}} & =\frac{\partial L_{n}}{\partial p_{n}}\frac{\partial p_{n}}{\partial w_{\;p}},\\ & =\frac{\partial L_{n}}{\partial p_{n}}⊙\varphi^{\prime }h_{n}^{T}. \end{array}$$

$\partial L_{n}/\partial w_{h}$ is more tricky, so we will go step by step. We already know that

4.3 $$\frac{\partial L_{n}}{\partial w_{h}}=\frac{\partial L_{n}}{\partial p_{n}}\frac{\partial p_{n}}{\partial h_{n}}\frac{\partial h_{n}}{\partial w_{h}}.$$

However, we see in figure 9 that **h**_*n*_ depends on **h**_*n*−1_, which depends on **h**_*n*−2_ (and so on). We also notice that all the hidden states depend on **w**_*h*_. We therefore rewrite equation (4.3) to make this explicit,

$$\begin{array}{rl} \frac{\partial L_{n}}{\partial w_{h}} & =\frac{\partial L_{n}}{\partial p_{n}}\frac{\partial p_{n}}{\partial h_{n}}\sum_{\;j=1}^{n}\frac{\partial h_{n}}{\partial h_{\;j}}\frac{\partial h_{\;j}}{\partial w_{h}},\\ & =\frac{\partial L_{n}}{\partial p_{n}}\frac{\partial p_{n}}{\partial h_{n}}\sum_{\;j=1}^{n}\left(\prod_{i=j+1}^{n}\frac{\partial h_{i}}{\partial h_{i-1}}\right)\frac{\partial h_{\;j}}{\partial w_{h}}. \end{array}$$

We can now substitute in some known values,

4.4 $$\frac{\partial L_{n}}{\partial w_{h}}=\frac{\partial L_{n}}{\partial p_{n}}⊙\varphi^{\prime }h_{n}^{T}\sum_{\;j=1}^{n}\left(\prod_{i=j+1}^{n}\phi^{\prime }w_{h,i}^{T}\right)\phi^{\prime }h_{\;j-1}^{T}.$$

Finally, $\partial L_{n}/\partial w_{x}$ is derived in the same way as $\partial L_{n}/\partial w_{h}$

4.5 $$\begin{array}{rl} \frac{\partial L_{n}}{\partial w_{x}} & =\frac{\partial L_{n}}{\partial p_{n}}\frac{\partial p_{n}}{\partial h_{n}}\sum_{\;j=1}^{n}\left(\prod_{i=j+1}^{n}\frac{\partial h_{i}}{\partial h_{i-1}}\right)\frac{\partial h_{\;j}}{\partial w_{x}},\\ & =\frac{\partial L_{n}}{\partial p_{n}}⊙\varphi^{\prime }h_{n}^{T}\sum_{\;j=1}^{n}\left(\prod_{i=j+1}^{n}\phi^{\prime }w_{h,i}^{T}\right)\phi^{\prime }x_{\;j}^{T}. \end{array}$$

With $\partial L_{n}/\partial w_{\;p}$, $\partial L_{n}/\partial w_{h}$ and $\partial L_{n}/\partial w_{x}$ in hand we can apply the same update rule shown in equation (2.5).

Aside from many-to-one encoding, RNNs can produce many predictions given many inputs, or act similarly to an MLP and produce one or many outputs given a single input. We will discuss the application of recurrent neural networks to astronomical data in §5, after we introduce gated recurrent neural networks.

### 4.3. Sidestepping the vanishing gradient problem

In the early 1990s, researchers identified a major issue with the training of deep neural networks through backpropagation. Hochreiter first formally examined the ‘vanishing gradient’ problem in their diploma thesis (Hochreiter [98], see also later work by Bengio *et al.* [99]). Owing to the vanishing gradient problem, it was widely believed that training very deep artificial neural networks from scratch via backpropagation was impossible. In this section, we will explore what the vanishing gradient problem is, and how contemporary end-to-end trained neural networks sidestep this issue.

First let us remind ourselves of the sigmoid activation function introduced in figure 6,

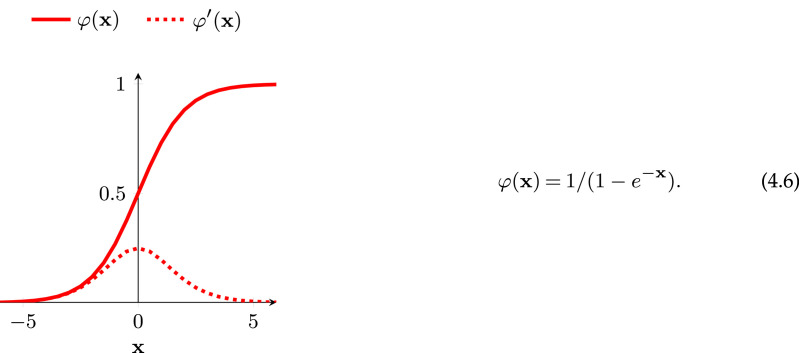
Equation (4.6) and its accompanying plot shows the output of a sigmoid function φ and its derivative φ′, when given an input **x**.

Now, let us revisit the weight update rule for the (*L* − *n*)th layer of a feedforward MLP (equation (2.4))

4.7 $$\frac{\partial L}{\partial w_{L-n}}=\frac{\partial L}{\partial p_{L}}⊙\underset{\lim_{n\to \infty }\prod_{i=1}^{n}\varphi_{L-i}^{\prime }w_{L-i}^{T}=\;0}{\underbrace{\left(\prod_{i=1}^{n}\varphi_{L-i}^{\prime }w_{L-i}^{T}\right)}}\;(\varphi_{L-n}^{\prime }p_{L-n-1}^{T}).$$

If φ′ is typically less than one (as in equation (4.6) and most other saturating nonlinearities) the product term in the above equation becomes an issue. In that case, we can see that the product rapidly goes to zero as *n* (the number of layers) becomes large.^16^ If we study equation (4.4), we can see the same problem also plagues RNNs as we backpropagate through hidden states

4.8 $$\frac{\partial L_{n}}{\partial w_{h}}=\frac{\partial L_{n}}{\partial p_{n}}⊙\varphi^{\prime }h_{n}^{T}\sum_{\;j=1}^{n}\underset{\lim_{n\to \infty }\prod_{i=j+1}^{n}\phi^{\prime }w_{h,i}^{T}=\;0}{\underbrace{\left(\prod_{i=j+1}^{n}\phi^{\prime }w_{h,i}^{T}\right)}}\phi^{\prime }h_{\;j-1}^{T}.$$

Let us solidify this issue by reminding ourselves of equation (2.5)—the weight update rule for a network trained through backpropagation

4.9 $$w_{\text{next}}=w-\eta \frac{\partial L}{\partial w}.$$

Combining equation (4.9) and the limits defined in equations (4.7) and (4.8) results in the below weight update rule in the limit *n* → ∞.

4.10 $$\lim_{n\to \infty }w_{\text{next}}=w.$$

Equation (4.10) shows that learning via backpropagation slows as we move deeper into the network. This problem once again caused a loss of faith in the connectionist model, ushering in the second AI winter. It took until 2012 for a new boom to begin. In the following three subsections, we will explore some of the proposed partial solutions to the vanishing gradient problem and show how they came together to contribute to the current deep learning boom.

#### 4.3.1. Non-saturating activation functions

We can see in equations (4.8) and (4.7) that if φ′ = 1 then the product term does not automatically go to zero or infinity. If this is the case, why not simply design our activation function around this property? The rectified linear unit (ReLU; [46,47]) is an activation function that does precisely this,^17^

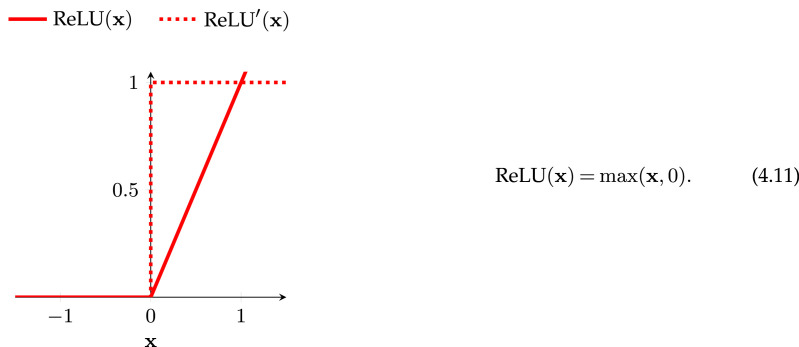


The gradient of ReLU is unity if the inputs are above zero, exactly the property we needed to mitigate the vanishing gradient problem. Similar non-saturating activation functions also share the ReLU gradient’s useful property, see for example the exponential linear unit, Swish and Mish functions in figure 6.

#### 4.3.2. Graphics processing unit acceleration

If we can speed up training, we can run an inefficient algorithm (such as backpropagation through saturating activations) to completion in less time. One way to speed up training is by using hardware that is specifically suited to the training of neural networks. Graphics processing units (GPUs) were originally developed to render video games and other intensive graphical processing tasks. These rendering tasks require a processor capable of massive parallelism. We have seen in the previous sections that neural networks trained through backpropagation also require many small weight update calculations. With this in mind, it is natural to try to accelerate deep neural networks using GPUs.

**Figure 10.**
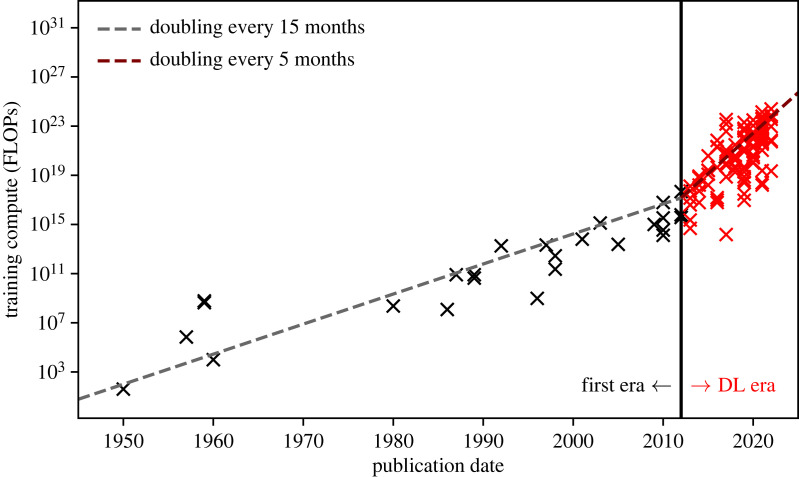
If we plot the total number of floating point operations (FLOPs) required to train a neural network model, and compare it with the model’s publication date, we can see a change in trend at around 2012. This corresponds to the popularization of GPU-accelerated training of very deep neural networks, with 2012 demarcating AI’s ‘Deep Learning Era’ and the beginning of astronomy’s second wave of connectionism (§5). Data are taken from Sevilla *et al.* [111].

In 2004, Oh & Jung [102] were the first to use GPUs to accelerate an MLP model, reporting a 20× performance increase on inference with an ‘ATI RADEON 9700 PRO’ GPU accelerated neural network. Shortly after, Steinkrau *et al.* [103] showed that backpropagation can also benefit from GPU acceleration, reporting a threefold performance increase in both training and inference. These two breakthroughs were followed by a flurry of activity in the area (e.g. [104–107]), culminating in a milestone victory for GPU accelerated neural networks at ImageNet 2012. AlexNet [108] won the ImageNet classification and localization challenges [109], scoring an unprecedented top-5 classification error of 16.4%, and a single object localization error of 34.2%. In both challenges, AlexNet scored over 10% better than the models in second place. Sutskever & Hinton’s winning network was a CNN [46] trained through backpropagation [40,93], with ReLU activation [47] and dropout [110] as a regularizer.^18^ The performance increase afforded by GPU-accelerated training enabled the network to be trained from scratch via backpropagation in a reasonable amount of time. The discovery that it is possible to train a neural network from scratch by using readily available hardware ultimately resulted in the end of connectionism’s second winter, and ushered in the Cambrianesque deep learning explosion of the mid-to-late 2010s and the 2020s (figure 10).

#### 4.3.3. Gated recurrent neural networks and residual networks

**Figure 11.**
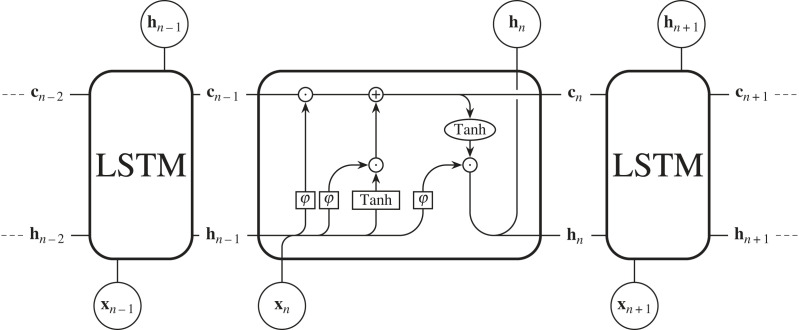
A set of sequential data **x**_*n*_ is input into an LSTM network. Inside the cell ○ denotes elementwise operations and □ denotes neuronal layers. φ is the sigmoid activation function, and Tanh is the hyperbolic tangent activation function. ⊕ is an elementwise addition, ⊙ is the Hadamard product, and line mergers are concatenations. **c**_*n*_ is the cell state, and **h**_*n*_ is the hidden state.

The long short-term memory unit (LSTM, [112,113])^19^ mitigates the vanishing gradient problem by introducing a new hidden state, the ‘cell state’ (**c**_*n*_), to the standard RNN architecture. This cell state allows the network to learn long-range dependencies, and we will show why this is the case via a brief derivation.^20^ First, as always, let us study figure 11 and write down the forward pass equation for updating the cell state

$$c_{n}=f\;(c_{n-1},h_{n-1},x_{n})+g(h_{n-1},x_{n}),$$

where $f\;(c_{n-1},h_{n-1},x_{n})=c_{n-1}⊙\varphi (h_{n-1},x_{n})$. For brevity we define φ_*n*_ = φ(**h**_*n*−1_, **x**_*n*_).

Like the RNN case (equations (4.4) and (4.5)), we will need to find ∂**c**_*n*_/∂**c**_*n*−1_ to calculate ∇L. Therefore,

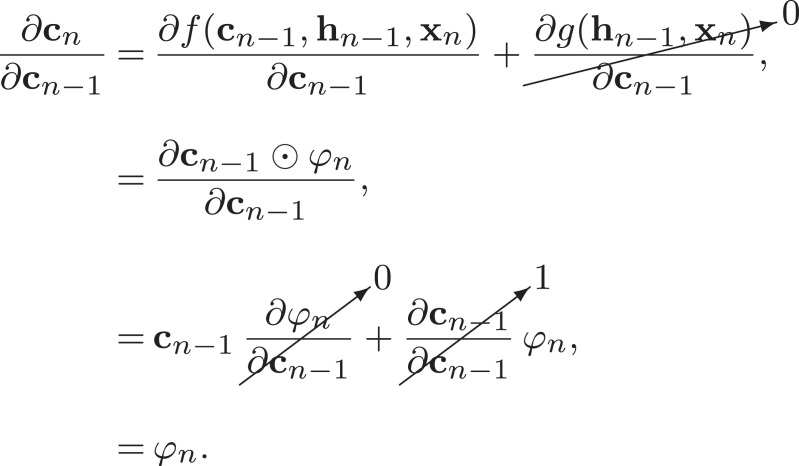
Thus, if we want to backpropagate to a cell state deep in the network, we must calculate

4.12 $$\frac{\partial c_{n}}{\partial c_{N}}=\prod_{i=1}^{n-N}\varphi_{i},\;n>N.$$

The product term above does not depend on the derivative of a saturating activation function, and so does not automatically vanish as *N* goes to ∞. This means that a gradient signal can be carried through the LSTM cell state without losing amplitude and vanishing.^21^

**Figure 12.**
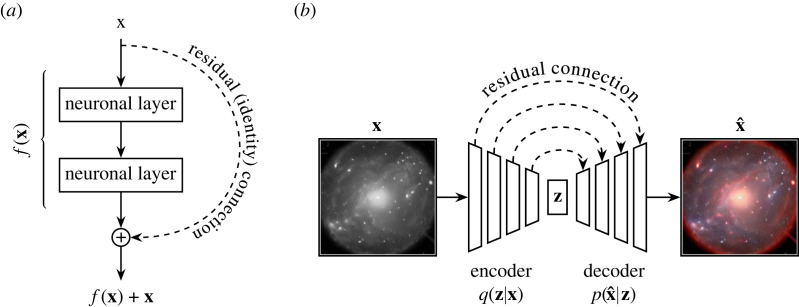
Panel (*a*) shows the residual connection as originally introduced in He *et al.* [119]. Panel (*b*) shows an application of the residual connection to an autoencoder-like U-Net architecture [120], in this case colourizing an astronomical object. Here, **z** is a compressed shared representation of **x** and $\hat{x}$.

We can use a technique derived from the LSTM to solve our vanishing gradient problem for deep feedforward neural networks (as studied in §2.2). Srivastava *et al.* [118] do this by applying the concept of the LSTM’s cell state to their deep convolutional ‘highway network’. The highway network uses gated connections to modulate the gradient flow back through neuronal layers. Later work by He *et al.* [119] introduces the residual network (ResNet) by taking a highway network and simplifying its connections. They apply an elementwise addition (or ‘residual connection’) in place of the highway network’s gated connection (figure 12*a*). One can go even further with residual connections, as Ronneberger *et al.* [120] demonstrate with their U-Net model. The U-Net combines residual connections with an autoencoder-like architecture (figure 12*b*). The U-Net has gone on to become the *de facto* network for many tasks that require an input and output of the same size (such as segmentation, colourization and style transfer).

### 4.4. Translation, attention and transformers

**Figure 13.**
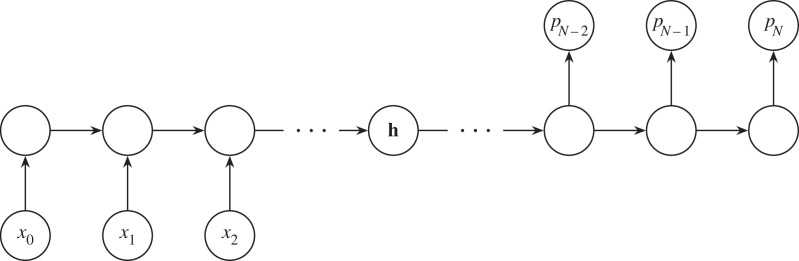
A sequence to sequence (Seq2Seq; [116]) model. A sequence **x** is input into a GRNN. The final hidden state (**h**) of the input network is then passed into a second GRNN. The second GRNN then unrolls to predict an output sequence **p**. Owing to the hidden state acting as an intermediary, **x** and **p** need not be of equal length.

Theoretically, gated RNNs (GRNNs) such as the LSTM can learn very long-range dependencies (see equation (4.12) and its accompanying text). In practice, GRNNs tend to forget information about distant inputs. This is because the GRNN lacks unmediated access to inputs beyond the immediate antecedent as a consequence of its recurrent architecture. The problem is especially apparent in neural machine translation tasks that require knowledge of an entire sequence to produce an output, such as language to language translation. Figure 13 shows such a sequence to sequence (Seq2Seq; [116]) model. Seq2Seq translates between two sets of sequential data by sharing a hidden state between two GRNN units. In figure 13, we can see that the shared information is bottlenecked by the hidden state. Therefore, to resolve the GRNN ‘forgetting problem’ we must find a way to avoid any recursion, or serial processing of input and output. We can do this by providing the neural network access to all input while it is calculating an output. This was the primary motivation behind the transformer architecture [117,121].

**Figure 14.**
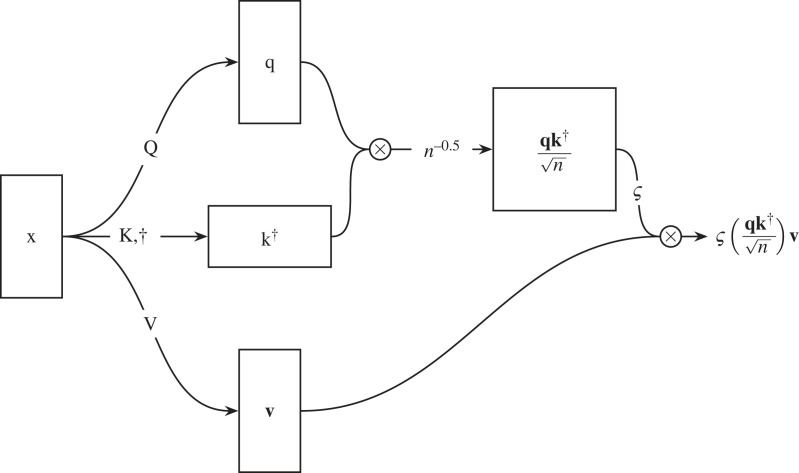
An input (**x**) is fed into a self-attention mechanism. The weights used to produce the query (**q**), key (**k**) and value (**v**) matrices are learnt via backpropagation. Here the learnt weights are denoted as the capitalized versions of their child matrices. **q** and **k** are normalized and multiplied together, and a softmax nonlinearity (ς) is applied. Finally, **v** is multiplied with output of the upper path and the final output is fed forward to the next neuronal layer. ⨂ denotes a matrix multiplication.

Modern transformer architectures consist of a series of self-attention layers interspersed with other layer types.^22^ Self-attention as described in Vaswani *et al.* [117] is shown in figure 14. Intuitively, it captures the relationships between quanta within a data input. To perform self-attention, we first take an input sequence

$$x=\left[\begin{array}{cccc} x_{1} & x_{2} & \cdots & x_{n} \end{array}\right],$$

where **x** can be any sequence, such as a sentence, a variable star’s time series, or an unravelled galaxy image.^23^ This sequence has a maximum length (*n*) that must be defined at train time, but we can process shorter sequences by masking out any surplus values so that they do not affect the loss. Here we will follow the literature and refer to [*x*_1_, …, *x*_*n*_] as tokens. As we can see in figure 14, the input is passed through a trainable pair of weight matrices **Q** (or ‘query’) and **K** (or ‘key’). The output matrices **q** and $k^{†}$ are then multiplied together to yield

4.13 $$(Q\cdot x){\left(K\cdot x\right)}^{†}=qk^{†}=\left[\begin{array}{cccc} Q_{1}x_{1}K_{1}x_{1} & Q_{1}x_{1}K_{2}x_{2} & \cdots & Q_{1}x_{1}K_{n}x_{n}\\ Q_{2}x_{2}K_{1}x_{1} & Q_{2}x_{2}K_{2}x_{2} & \cdots & Q_{2}x_{2}K_{n}x_{n}\\ \vdots & \vdots & \ddots & \vdots \\ Q_{n}x_{n}K_{1}x_{1} & Q_{n}x_{n}K_{2}x_{2} & \cdots & Q_{n}x_{n}K_{n}x_{n} \end{array}\right].$$

We can see that equation (4.13) describes the relationships between tokens within **x**. For example, if *x*_1_ is similar semantically to *x*_2_, we would expect *Q*_1_*x*_1_*K*_2_*x*_2_ and *Q*_2_*x*_2_*K*_1_*x*_1_ to have a high value. We then normalize $qk^{†}$ to mitigate vanishing gradients (see footnote 16) and apply a softmax nonlinearity so that the maximum weighting (or similarity) is one and the similarity values sum to unity.

Meanwhile, the input sequence **x** is passed through the neuronal layer **V**, resulting in a weighted representation **v**,

$$V\cdot x=v=\left[\begin{array}{cccc} V_{1}x_{1} & V_{2}x_{2} & \cdots & V_{n}x_{n} \end{array}\right].$$

**v** is multiplied with the similarity matrix $\varsigma (qk^{†}/\sqrt{n})$. This process weighs similar tokens within the sequence higher, increasing their relative importance in later neuronal layers.

We will use an astronomical example to solidify our understanding of the self-attention mechanism. Let us assume that our self-attention mechanism is attending to a natural language caption describing a galaxy’s morphology that has been provided by a citizen scientist. The caption could be something like:

x=A barred galaxy with five spiral arms,

with each word acting as a separate token. Let us imagine that we put this prompt into our self-attention mechanism,

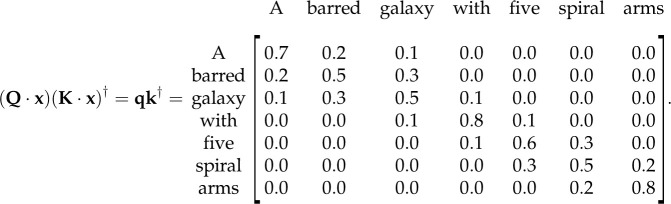

**Figure 15.**
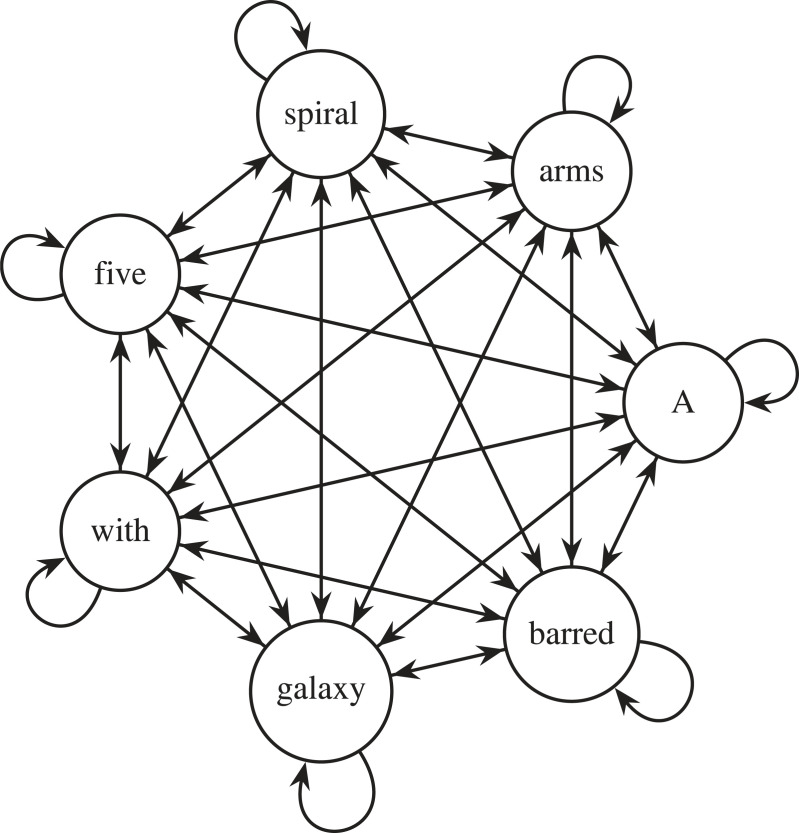
We can think of $qk^{†}$ within self-attention as a graph of relationships between a prompt and itself. Each of the edges in this graph represents the weight shared between a pair of tokens in the input sequence.

We can see that in the above matrix higher values have been assigned to pairs of words that are more closely related within the sentence. For example, the weight between ‘barred’ and ‘galaxy’ is relatively high (0.3), as the term ‘barred’ describes a feature of galaxy. Similarly, the weight between ‘five’ and ‘spiral’ is also high (0.3), as these words together define the number of spiral arms in the galaxy. Conversely, lower weights have been assigned to word pairs that are less related, such as ‘*A*’ and ‘*with*’ (0.0). As shown in figure 15, one can think of these relationships between tokens within our sequence as a learnt mathematical graph.^24^ Now that we have calculated $qk^{†}$, we can use this matrix to weigh our example sentence as shown in figure 14. This weighting gives the subsequent layers in our neural network an awareness of the relationships between the tokens in our sequence.

## 5. Astronomy’s second wave of connectionism

Compared with classical connectionist approaches^25^ deep learning as outlined in §4 does not require an extraction of emergent parameters to train its models. CNNs in particular are well suited to observing raw information within image-based data. Likewise, RNNs are well suited to observing the full raw information within a time series. Astronomy is rich with both types of data, and in this section we will review the history of the application of CNN, RNN and transformer models to astronomical data.

### 5.1. Convolutional neural network applications

It did not take long after Krizhevsky *et al.* [108] established CNNs as the de facto image classification network for astronomers to take notice: in 2014, they were applied in the search for pulsars [129] as part of an ensemble of methods. Zhu *et al.* [129] found that their ensemble was highly effective, with 100% of their test set pulsar candidates being ranked within the top 961 of the 90 008 test candidates. Shortly after, Hála [130] described the use of one-dimensional CNNs for a ternary classification problem. They found that their model is capable of classifying one-dimensional spectra into quasars, galaxies and stars to an impressive accuracy. CNNs have also been extensively used in galaxy morphological classification. First on the scene was Dieleman *et al.* [131]. They used CNNs to classify galaxy morphology parameters as defined in the Galaxy Zoo dataset [132] from galaxy imagery. They observed their galaxies via the SDSS, and found a 99% consensus between the Galaxy Zoo labels, and the CNN classifications. Huertas-Company *et al.* [133] showed that the CNN introduced in Dieleman *et al.* [131] is equally applicable to the morphological classification of galaxies in the CANDELS fields [134]. Likewise, Aniyan & Thorat [135] showed that CNNs are capable of classifying radio galaxies. The combined work of Dieleman *et al.* [131], Huertas-Company *et al.* [133] and Aniyan & Thorat [135] confirms that CNNs are equally applicable to visually dissimilar surveys, with little-to-no modification. Looking a little further afield, Wilde *et al.* [136] used a deep CNN model to classify simulated lensing events. They also applied some interpretability techniques to their data, using occlusion mapping [137], gradient class activation mapping [138] and Google’s DeepDream to prove that the CNN was indeed classifying via observing the gravitational lenses. Alternative CNN models have also been used, such as the U-Net (figure 12*b*). The U-Net was initially developed to segment biological imagery [120]. Its first use in astronomy was related: Akeret *et al.* [139] used a U-Net [120] CNN to isolate via segmentation, and ultimately remove, radio frequency interference from radio telescope data. Likewise, Berger & Stein [140] used a three-dimensional U-Net (V-Net; [141]) to predict and segment out galaxy dark matter haloes in simulations, and Aragon-Calvo [142] used a V-Net to segment out the cosmological filaments and walls that make up the large-scale structure of the Universe. Hausen & Robertson [143] demonstrate that a U-Net is capable of performing pixelwise semantic classification of objects in HST/CANDELS imagery, thus proving that U-Nets are capable of useful work directly within large imaging surveys, particularly in the deblending of overlapping objects, which is a perennial challenge in deep imaging. The U-Net in Lauritsen *et al.* [144] is used to super-resolve simulated submillimetre observations. They found that the U-Net could successfully do this when using a loss comprising the L1 loss and a custom loss that measures the distance between predicted and ground truth point sources. Choma *et al.* [145] were the first to demonstrate that graph convolutional neural networks (GCNNs) are useful within astronomical context. They showed that their three-dimensional GCNN could classify signals from the IceCube neutrino observatory, and found that it outperformed both a classical method, and a standard three-dimensional CNN. Villanueva-Domingo *et al.* [146,147] demonstrated that EdgeNet—a class of GCNN—can estimate halo masses when given the positions, velocities, stellar masses and radii of the host galaxies [148]. The authors also demonstrated that EdgeNet can estimate the halo masses of both Andromeda and the Milky Way. We must conclude from the studies described in this subsection that CNNs are effective classifiers and regressors of image-based astronomical data.

### 5.2. Recurrent neural network applications

RNNs were first applied in astronomy very close to home; Aussem *et al.* [149] predicted atmospheric seeing for observations from the European Southern Observatory's Very Large Telescope, and the prediction of geomagnetic storms given data on the solar wind was also explored in the mid-to-late 1990s and early 2000s ([150,151] and other work from the same group; [152]).

The first use of RNNs for classification in astronomy was carried out in a prescient study by Brodrick *et al.* [153]. They describe the use of an RNN-like Elman network [154]. Their RNN was tasked with the search for artificially generated narrowband radio signals that resemble those that may be produced by an extraterrestrial civilization. They found that their model had a test set accuracy of 92%, suggesting that RNNs could be a useful tool in the search for extraterrestrial intelligence. More than a decade after Brodrick *et al.* [153], Charnock & Moss [155] used an LSTM (figure 11) to classify simulated supernovae. They describe two classification problems. One, a binary classification between type-Ia and non-type-Ia supernovae, and the other a classification between supernovae types I, II and III. For their best performing model, they report an accuracy of more than 95% for their binary classification problem, and an accuracy of over 90% for their trinary classification. This study cemented the usefulness of RNNs for classification problems in astronomy. Charnock & Moss [155] were followed by numerous projects studying the use of RNNs for classification of time-series astronomical data. A non-exhaustive list of modern RNN use in astronomy includes: stochastically sampled variable star classification [156], exoplanet instance segmentation [157], variable star/galaxy sequential imagery classification [158] and gamma ray source classification [159]. We must conclude from these studies that RNNs are effective classifiers of astronomical time series, provided that sufficient data are available.

Of course, recurrent networks are not limited to classification; they can also be used for regression problems. First, Weddell & Webb [160] successfully used an echo state network [161] to predict the point spread function of a target object in a wide field of view. Capizzi *et al.* [162] used an RNN to inpaint missing NASA Kepler time series data for stellar objects. They found that their model could recreate the missing time series to an excellent accuracy, suggesting that the RNN could internalize information about the star it was trained on. As in the classification case, research into the use of RNNs for regression problems picked up massively in the late 2010s, and here we will highlight a selection of these studies that represent the range of RNN use cases. Shen *et al.* [163] used both an LSTM and an autoencoder-based RNN to denoise gravitational wave data, and Morningstar *et al.* [164] used a recurrent inference machine to reconstruct gravitationally lensed galaxies. Liu *et al.* [165] used an LSTM to predict solar flare activity. From these studies, similarly to the classification case above, we can once again conclude that RNNs are effective regressors of astronomical time series.

RNNs have also been used in cases that are a little more unconventional. For example, Kügler *et al.* [166] used an autoencoding RNN (specifically an echo state network) to extract representation embeddings of variable main sequence stars. They find that these embeddings capture some emergent properties of these variable stars, such as temperature and surface gravity, suggesting that clustering within the embedding space could result in semantically meaningful variable star classification. We will revisit this line of research when we explore representation learning within astronomy in detail in §8. An example of more drastic cross-pollination between ideas within deep learning and those within astronomy is Smith *et al.* [167]. They use an encoder–decoder network comprising a CNN encoder and RNN decoder to predict surface brightness profiles of galaxies. This class of neural network was previously used extensively within natural language image captioning, and by treating surface brightness profiles as ‘captions’ their model was capable of prediction over 100× faster than the previous classical, human-agent-based method.

### 5.3. Transformer applications

Although initially used for natural language, transformers have also been adapted for use in imagery, first by Parmar *et al.* [168], and also in Dosovitskiy *et al.* [18]. To the best of our knowledge, transformers have not yet been applied to astronomical imagery, but they have started to find use in time-series astronomy. Donoso-Oliva *et al.* [169] used BERT [123] to generate a representation space for light curves in a self-supervised manner. Morvan *et al.* [170] used an encoding transformer to denoise light curves from the Transiting Exoplanet Survey Satellite (TESS, [171]) and show that the denoising surrogate task results in an expressive embedding space. Pan *et al.* [172] also use a transformer model to analyse light curves for exoplanets. Transformers have taken the fields of natural language processing and computer vision by storm (§9), and so if we extrapolate from trends in other fields we expect to see many more examples of transformers applied to astronomical use cases in the near future. We will revisit the transformer architecture in the context of foundation models ([173] and references therein) and their possible future astronomical applications in §9.

### 5.4. A problem with supervised learning

Supervised learning requires a high-quality labelled dataset to train a neural network. In turn, these datasets require laborious human intervention to create, and so supervised data is in short supply. One can avoid this issue by prompting the deep learning model to gather semantic information from entirely unlabelled data. This learnt semantic information can then be accessed through a hidden descriptive ‘latent space’, and then used for downstream tasks like data generation, classification and regression. Indeed, all of the networks described previously in this review can be repurposed for non-supervised tasks, and in §§6 and 7 we will explore some deep learning frameworks that do not require supervision.

## 6. Deep generative modelling

**Figure 16.**
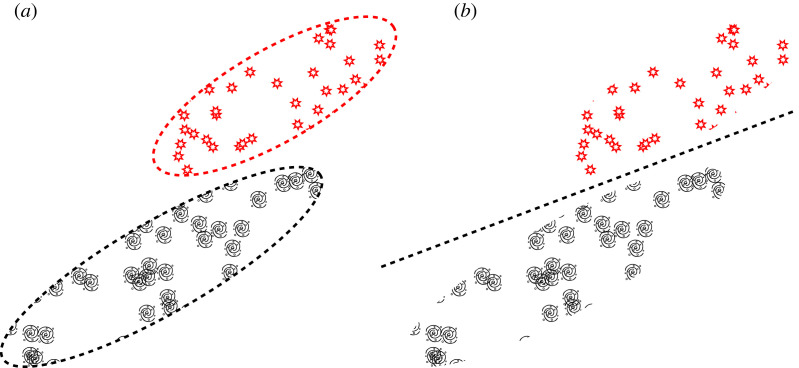
Here we show a possible latent space representation of a set of galaxies and a set of stars. A latent (or embedding) space is a compressed representation of a set of objects where similar objects are clustered closer together than dissimilar objects. While this space is often highly dimensional, here we project our latent space onto two dimensions for visualization purposes. In (*a*), we see a generative model attempting to learn the probability distributions of the latent representation of a dataset that contains a set of galaxies and a set of stars. In (*b*), we see a discriminative model attempting to learn the boundary that separates the star and galaxy types.

In this section, we discuss generative modelling within the context of astronomy. Unlike discriminative models, generative models explicitly learn the distribution of classes in a dataset (figure 16). Once we learn the distribution of data, we can use that knowledge to generate new synthetic data that resembles that found in the training dataset. In the following subsections, we will explore in detail three popular forms of deep generative model: the variational autoencoder (§6.1), the generative adversarial network (§6.2) and the family of score-based (or diffusion) models (§6.3). Finally, in §8 we discuss applications of deep generative modelling in astronomy.

### 6.1. (Variational) autoencoders

**Figure 17.**
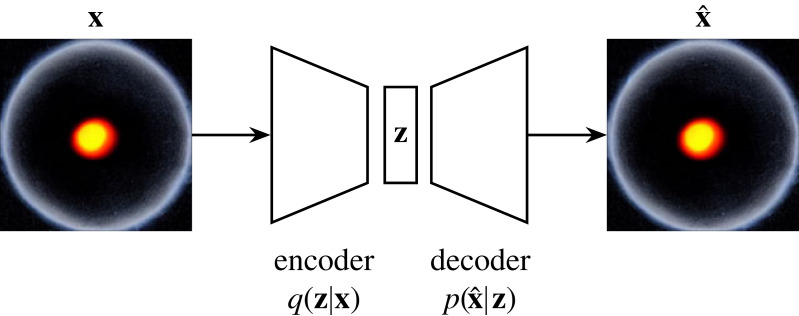
An autoencoder [174] attends to an image of a black hole. **z** is a latent vector and **x** is a sample from a training set. The encoder, *q* learns to encode the incoming data into a latent vector while the decoder *p* takes as input **z** and attempts to recreate **x**.

Autoencoders have long been a neural network architectural staple. In a sister paper to backpropagation’s popularizer, Rumelhart *et al.* [174] demonstrate backpropagation within an autoencoder. Figure 17 demonstrates the basic neural network autoencoder architecture. An autoencoder is tasked with recreating some input data, squeezing the input information (**x**) into a bottleneck latent vector (**z**) via a neural network q(z|x). **z** is then expanded to an imitation of the input data ($\hat{x}$) by a second neural network $p(\hat{x}|z)$. The standard autoencoder is trained via a reconstruction loss; $L_{R}(x,\hat{x})$, where $L_{R}(x,\hat{x})$ measures the difference in pixelspace between **x** and $\hat{x}$.

**Figure 18.**
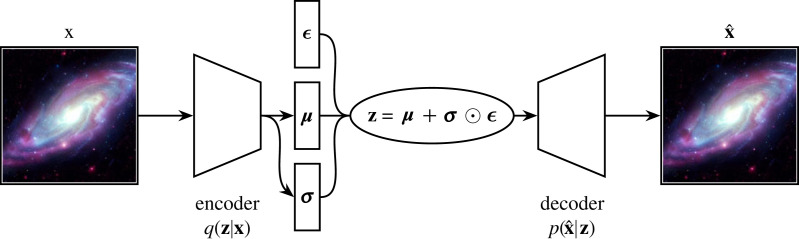
A variational autoencoder [175] operates on a spiral galaxy. **z** is a latent vector and **x** is a sample from the training set. The encoder, *q* learns to compress the incoming data into a latent vector that encodes the normal distribution. The decoder *p* takes as input **z** and attempts to recreate **x**.

Naively, one would think that once trained, one could ‘just’ sample a new latent vector, and produce novel imagery via the decoding neural network $p(\hat{x}|z)$. We cannot do this, as autoencoders trained purely via a reconstruction loss have no incentive to produce a smoothly interpolatable latent space. This means we can use a standard autoencoder to embed and retrieve data contained in the training set, but cannot use one to generate new data. To generate new data we require a smooth latent space, which variational autoencoders (VAEs, figure 18) produce by design [175].

A VAE differs from the standard autoencoder by enforcing a spread in each training set samples’ latent vector. We can see in figure 18 how this is done; instead of directly predicting **z** the encoder *q* predicts two vectors, ***μ*** and ***σ***. **z** is then sampled stochastically via the equation

6.1 z=μ+σ⊙ϵ,

where ⊙ is the Hadamard product, and ϵ is noise generated externally to the neural network graph.^26^ This spread results in similar samples overlapping within the latent space, and therefore we end up with a smooth latent space that we can interpolate through. However, currently there is no incentive for the neural network to provide a coherent, compact global structure in the latent space. For that we require a regularization term in the loss. This regularization is provided via the Kullback–Leibler (KL) divergence, which is a measure of the difference between two probability distributions. A standard VAE uses the KL divergence to push the latent distribution towards the standard normal distribution, incentivizing a compact, continuous latent space. Hence, the final VAE loss is a combination of the reconstruction loss and KL divergence:

6.2 $$L_{\text{VAE}}=L_{R}(x,\hat{x})+\text{KL}(q(x|z)\|\rho ),$$

where *ρ* is some prior. In a standard VAE ρ=N(0,1).

In practice, VAEs are able to generate smooth and coherent samples, as they model the data distribution explicitly, which also means that we can perform latent space arithmetic on the latent vector—such as interpolation, reconstruction and anomaly detection [175]. Their explicit learning of the latent vector (**z**) means that they can trivially be repurposed for semi-supervised, self-supervised and supervised downstream tasks by manipulating **z** [176,177]. However, the quality of samples generated by VAEs is lower than that of generative adversarial networks or score-based generative models [178]. This reduction in quality is due to the VAE’s simple posterior *q*(**z**|**x**), but one can mitigate this shortcoming by iteratively approaching a more complex posterior.^27^ To regularize the latent space, VAEs require an assumption of the prior distribution which requires some knowledge of the dataset, although often this can be set as ‘just’ a normal distribution as shown in equation (6.2).

### 6.2. Generative adversarial networks

Generative adversarial networks (GAN, [183]) can be thought of as a minimax game between two competing neural networks. If we anthropomorphize, we can gain an intuition for how a GAN learns: let us imagine an art forger and an art critic. The forger wants to paint paintings that are similar to famous expensive works, and needs to fool the critic when selling these paintings. Meanwhile, the critic wants to ensure that no reproductions are sold, and so they need to accurately determine whether any painting is an original or a reproduction. At first, our forger is a poor painter, and so the critic can easily identify our forger’s works. However, the forger learns from the critic’s choices and produces more realistic paintings. As the forger’s paintings improve, the critic also learns better methods for detecting forgeries. This minimax game incentivizes the critic to keep improving their classifications, and the forger to keep improving their painting. If this continues, we get to a point where the forger’s works are indiscernible from the real thing—the forger has learnt to perfectly mimic the dataset! In a GAN, we name the critic the discriminator (*D*), and we name the forger the generator (*G*).

**Figure 19.**
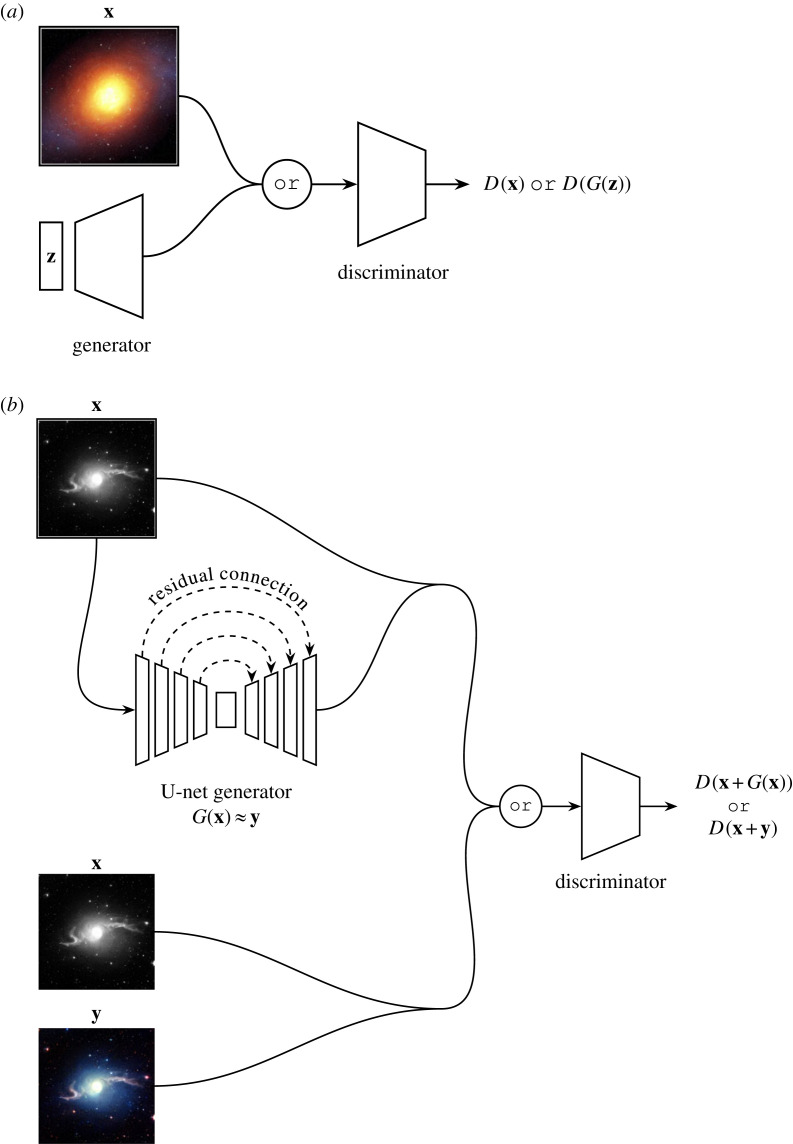
The GAN and Pix2Pix models. (*a*) A typical GAN according to Goodfellow *et al.* [183]. **z** is a noise vector, and **x** is a sample from the training set. The discriminator learns to classify the incoming images as either fake or real, and the generator learns to fool the discriminator by producing realistic fakes. (*b*) A Pix2Pix-like model with a U-Net generator [120,184]. The discriminator learns to classify the incoming image tuples as either fake or real. Meanwhile, the generator learns to fool the discriminator by approximating the colourization function mapping **x** → **y**. Line mergers denote channel-wise concatenations.

In Goodfellow *et al.*’s original GAN formulation (figure 19*a*), *G* and *D* are neural networks (typically CNNs, although other architectures can be used) that compete during training in a minimax game where *G* aims to maximize the probability of *D* mispredicting that a generated datapoint is sampled from the real dataset [183]. *G* takes as input a randomly sampled latent vector **z**, and outputs a synthetic datapoint *G*(**z**). *D* takes either this synthetic datapoint, or a real datapoint **x**, and outputs *D*(*G*(**z**)) or *D*(**x**). This output is the probability that the datapoint is drawn from the real dataset. To train the network, we can write the GAN adversarial loss like so

$$L_{D}=-(E_{x}[\log(D(x))]+E_{z}[\log(1-D(G(z)))])$$

and

$$L_{G}=E_{z}[\log(1-D(G(z)))],$$

where here we attempt to minimize both $L_{D}$ and $L_{G}$. In practice, we train the networks by alternating freezing the weights of *G* and backpropagating $L_{D}$, and then freezing the weights of *D* and backpropagating $L_{G}$ for each training batch. In this way, the networks’ weights are updated to follow $\nabla_{w}L_{G}$ and $\nabla_{w}L_{D}$ downwards until the distribution of *G*(**z**) closely resembles that of the real dataset. Once trained, *G* can be used to generate entirely novel synthetic data that closely resembles (but is not identical to) the training set data.

One can condition a GAN to guide the network towards a desired output image [185]. To do this, we alter the adversarial loss so that it is conditioned on a label **y**

$$L_{D}=-(E_{x}[\log(D(x|y))]+E_{z}[\log(1-D(G(z|y)))])$$

and

$$L_{G}=E_{z}[\log(1-D(G(z|y)))].$$

As an example, if we set **y** as the redshift of the galaxies in the training set, we could use a conditional GAN to guide the network to generate galaxies of a certain redshift. Furthermore, we are not restricted to conditioning single values; GANs can also be conditioned on entire images. In figure 19*b*, we see that the GAN adversarial loss can be used to translate between image domains [184]. In Isola *et al.*’s Pix2Pix model, the generator takes as input an image **x**, and attempts to produce a related image **y**. Meanwhile, the discriminator attempts to discern whether the (**x**, **y**) pair that it is given is sampled from the training set, or the generator. Otherwise, Pix2Pix is trained in the same way as the standard GAN.

GANs are capable of generating high-quality, sharp and realistic samples [186,187]. They have long been a sweetheart of the deep generative learning community, having been used for various state-of-the-art applications, such as data embedding (e.g. [188]), style transfer (e.g. [189]), super-resolution (e.g. [190]), and image inpaining and object removal (e.g. [191]). Unfortunately, however, GANs have some downsides. They are quite difficult to train; maintaining the balance between the generator and discriminator networks is challenging and requires careful fine-tuning [192]. *G* and *D* must work in tandem and one cannot overpower the other or learning will cease. One of the most famous symptoms of this imbalance is mode collapse, where *G* only generates a limited variety of samples that reliably fool *D*. This instability during training makes it quite a time-consuming task to find a stable network architecture if one is designing a GAN themselves. Finally, the GAN adversarial losses are relative and so are not representative of the image quality. This is not the case for the VAE and score-based generative model (SBGM) families of models.

### 6.3. Score-based generative modelling and diffusion models

Diffusion models were introduced by Sohl-Dickstein *et al.* [193] and were first shown to be capable of producing high-quality synthetic samples by Ho *et al.* [194]. Diffusion models are part of a family of generative deep learning models that employ denoising score matching via annealed Langevin dynamic sampling (first explored by Hyvärinen [195] and Vincent [196]. More recent work can be found in [194,197–200]). This family of SBGMs can generate imagery of a quality and diversity surpassing state-of-the-art GAN models [183], a startling result considering the historic disparity in interest and development between the two techniques [200–203]. SBGMs can super-resolve images [204,205], translate between image domains [206], separate superimposed images [207] and in-paint information [200,204].

**Figure 20.**
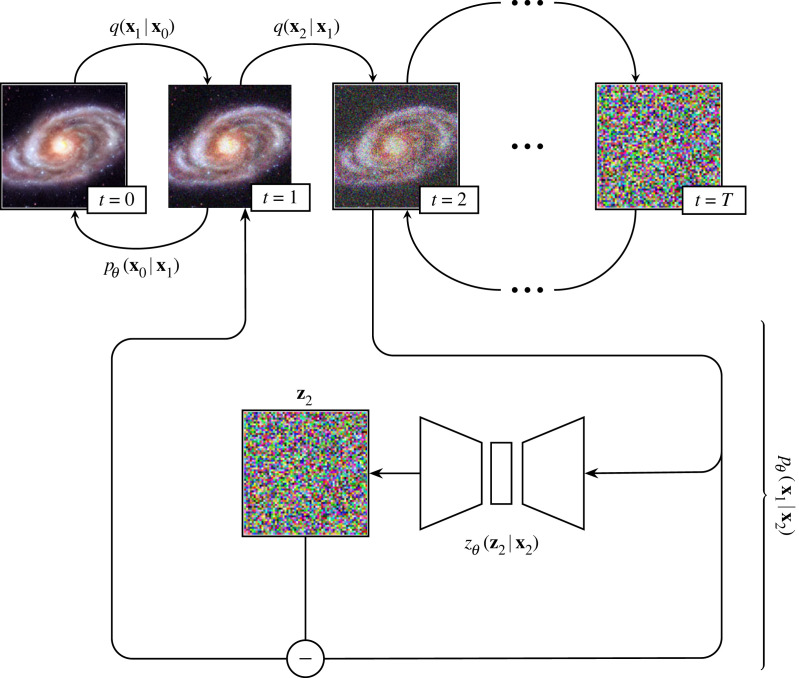
It is easy (and achievable without learnt parameters) to add noise to an image, but more difficult to remove it. Diffusion models attempt to learn an iterative removal process via training an appropriate neural network, $p_{\theta }(x_{t-1}|x_{t})$.

Diffusion models define a diffusion process that projects a complex image domain space onto a simple domain space. In the original formulation, this diffusion process is fixed to a predefined Markov chain *q*(**x**_*t*_|**x**_*t*−1_) that adds a small amount of Gaussian noise with each step. As figure 20 shows, this ‘simple domain space’ can be noise sampled from a Gaussian distribution $x_{T}∼N(0,1)$.

#### 6.3.1. Forward process

To slowly add Gaussian noise to our data, we define a Markov chain

$$q(x_{0\ldots T})=q(x_{0})\prod_{t=1}^{T}q(x_{t}|x_{t-1}),$$

where **x**_0_ is an image sampled from the training set. The amount of noise added per step is controlled with a variance schedule ${\left\{\beta_{t}\in (0,1)\right\}}_{t=1}^{T}$

6.3 $$q(x_{t}|x_{t-1})=N(x_{t};\sqrt{1-\beta_{t}}\;x_{t-1},\;\beta_{t}1).$$

This process is applied incrementally to the input image. Since we can define the above equation such that it only depends on **x**_0_ we can immediately calculate an image representation **x**_*t*_ for any *t* [194]. If we define *α*_*t*_ = 1 − *β*_*t*_ and ${\bar{\alpha }}_{t}=\prod_{i=1}^{t}\alpha_{i}$:

6.4 $$\begin{array}{rl} x_{t} & =\sqrt{\alpha_{t}}\;x_{t-1}+\sqrt{1-\alpha_{t}}\;z_{t-1}\\ & =\sqrt{\alpha_{t}\alpha_{t-1}}\;x_{t-2}+\sqrt{\left(1-\alpha_{t})+\alpha_{t}(1-\alpha_{t-1}\right)}\;{\bar{z}}_{t-2}\\ & =\sqrt{\alpha_{t}\alpha_{t-1}\alpha_{t-2}}\;x_{t-3}+\sqrt{\left(1-\alpha_{t}\alpha_{t-1})+\alpha_{t}\alpha_{t-1}(1-\alpha_{t-2}\right)}\;{\bar{z}}_{t-3}\\ & =\cdots \\ & =\sqrt{\bar{\alpha_{t}}}x_{0}+\sqrt{1-{\bar{\alpha }}_{t}}z, \end{array}$$

where $z_{t}∼N(0,1)$ and $\bar{z}$ is a combination of Gaussians. Plugging the above expression into equation (6.3) removes the **x**_*t*−1_ dependency and yields

6.5 $$q(x_{t}|x_{0})=N(x_{t};\sqrt{{\bar{\alpha }}_{t}}\;x_{0},\;(1-{\bar{\alpha }}_{t})1).$$

#### 6.3.2. Reverse process

Diffusion models attempt to reverse the forward process by applying a Markov chain with learnt Gaussian transitions. These transitions can be learnt via an appropriate neural network, $p_{\theta }$

$$p_{\theta }(x_{0\ldots T})=p(x_{T})\prod_{t=1}^{T}p_{\theta }(x_{t-1}|x_{t})$$

and

$$p_{\theta }(x_{t-1}|x_{t})=N(x_{t-1};\mu_{\theta }(x_{t},t),\Sigma_{\theta }(x_{t},t)).$$

While $\Sigma_{\theta }(x_{t},t)$ can be learnt (e.g. [201]), the Ho *et al.* [194] formulation fixes $\Sigma_{\theta }$ to an iteration-dependent constant $\sigma_{t}^{2}1$, where $\sigma_{t}^{2}=1-\alpha_{t}$.

By recognizing that diffusion models are a restricted class of hierarchical VAE,^28^ we see that we can train $p_{\theta }$ by optimizing the evidence lower bound (ELBO, introduced in [175]) that can be written as a summation over the KL divergences at each iteration step^29^

6.6 $$\begin{array}{rl} L_{\text{ELBO}}= & E_{q}[D_{\text{KL}}(q(x_{T}|x_{0})\|p(x_{T}))\\ & +\sum_{t>1}D_{\text{KL}}(q(x_{t-1}|x_{t},x_{0})\|p_{\theta }(x_{t-1}|x_{t}))+\log p_{\theta }(x_{0}|x_{1})]. \end{array}$$

In the Ho *et al.* [194] formulation, the first term in equation (6.6) is a constant during training and the final term is modelled as an independent discrete decoder. This leaves the middle summation. Each summand can be written as

6.7 $$L(\mu_{t},\mu_{\theta })=\frac{1}{2\sigma_{t}^{2}}\|\mu_{t}(x_{t},x_{0})-\mu_{\theta }(x_{t},t){\|}^{2},$$

where $\mu_{\theta }$ is the neural network’s estimation of the forward process posterior mean ***μ***_*t*_. In practice, it would be preferable to predict the noise addition in each iteration step (**z**_*t*_), as **z**_*t*_ has a distribution that by definition is centred about zero, with a well-defined variance. To this end, we can define $\mu_{\theta }$ as

6.8 $$\mu_{\theta }(x_{t},t)=\frac{1}{\sqrt{\alpha_{t}}}\left(x_{t}-\frac{1-\alpha_{t}}{\sqrt{1-{\bar{\alpha }}_{t}}}z_{\theta }(x_{t},t)\right),$$

and by combining equations (6.7) and (6.8) we get

6.9 $$\begin{array}{rl} L(z_{t},z_{\theta }) & =\frac{1}{2\sigma_{t}^{2}}\|\frac{1}{\sqrt{\alpha_{t}}}\left(x_{t}-\frac{1-\alpha_{t}}{\sqrt{1-{\bar{\alpha }}_{t}}}z_{t}\right)-\frac{1}{\sqrt{\alpha_{t}}}\left(x_{t}-\frac{1-\alpha_{t}}{\sqrt{1-{\bar{\alpha }}_{t}}}z_{\theta }(x_{t},t)\right){\|}^{2}\\ & =\frac{{\left(1-\alpha_{t}\right)}^{2}}{2\sigma_{t}^{2}\alpha_{t}(1-{\bar{\alpha }}_{t})}\|z_{t}-z_{\theta }(x_{t},t){\|}^{2}. \end{array}$$

Ho *et al.* [194] empirically found that a simplified version of the loss described in equation (6.9) results in better sample quality. They use a simplified version of equation (6.9) as their loss, and optimize to predict the noise required to reverse a forward process iteration step:

6.10 $$L(z_{t},z_{\theta })=\|z_{t}-z_{\theta }(x_{t},\;t){\|}^{2},\;\text{where}\;x_{t}=\sqrt{{\bar{\alpha }}_{t}}x_{0}+\sqrt{1-{\bar{\alpha }}_{t}}z_{t}.$$

By recognizing that $z_{t}=\sigma_{t}^{2}\nabla_{x_{t}}\log q(x_{t}|x_{t-1})$, we see that equation (6.10) is equivalent to denoising score matching over *t* noise levels [196]. This connection establishes a link between diffusion models and other SBGMs (such as [197,198,210]).

To run inference for the reverse process, one progressively removes the predicted noise $z_{\theta }$ from an image. The predicted noise is weighted according to a variance schedule

$$x_{t-1}=\frac{1}{\sqrt{\alpha_{t}}}\left(x_{t}-\frac{1-\alpha_{t}}{\sqrt{1-{\bar{\alpha }}_{t}}}\;z_{\theta }(x_{t},t)\right)+\sigma_{t}z.$$

If we take $p(x_{T})∼N(x_{T};0,1)$, we can use $p_{\theta }$ to generate entirely novel data that are similar—but not identical to—those found in the training set.

In practice, diffusion models are trained by sampling an integer value of t∼U(1,T), where *T* is a large value typically in the thousands. We then use equation (6.5) to sample an image **x**_*t*_ that has had noise added to it *t* times. The model then attempts to predict the exact noise required to reverse a forward iteration time step—that is, the output of a neural network^30^ of the form $z_{\theta }(z_{t}|x_{t-1})$. As shown in figure 20, we can estimate **x**_*t*_ by removing the predicted noise from **x**_*t*−1_. To optimize the model, **z**_*t*_ is compared via equation (6.10) with the actual noise required to reverse the forward iteration, and this is the loss that is reduced during training. For a detailed astronomical example with code, we direct the reader to Smith *et al.* [13].

#### 6.3.3. Denoising diffusion implicit models

Ho, Jain and Abbeel’s diffusion model performs inference at a rate orders of magnitude slower than single-shot generative models like the VAE (§6.1) or the GAN (§6.2). This is because diffusion models need to sequentially reverse every step in the forward process Markov chain. Reducing the inference time for diffusion models is an active area of research [199,211,212], and here we will review one proposed solution to the problem; the denoising diffusion implicit model (DDIM, [213]).

Song *et al.* ([213], §§3–4) propose the following reparametrization of equation (6.4):

$$\begin{array}{rl} x_{t-1} & =\sqrt{{\bar{\alpha }}_{t-1}}\;x_{0}+\sqrt{1-{\bar{\alpha }}_{t-1}-\sigma_{t}^{2}}\;z_{\theta }^{\left(t\right)}+\sigma_{t}z_{t}\\ & =\sqrt{{\bar{\alpha }}_{t-1}}\;\underset{x_{0}\;\text{prediction}}{\underbrace{\left(\frac{x_{t}-\sqrt{1-{\bar{\alpha }}_{t}}\;z_{\theta }^{\left(t\right)}}{\sqrt{{\bar{\alpha }}_{t}}}\right)}}+\underset{\text{vector towards}\;x_{t}}{\underbrace{\sqrt{1-{\bar{\alpha }}_{t-1}-\sigma_{t}^{2}}\;z_{\theta }^{\left(t\right)}}}+\underset{\text{noise}}{\underbrace{\sigma_{t}z_{t}}}, \end{array}$$

where (*t*) is noted as a superscript to denote the output of the neural network $z_{\theta }$ at time step *t*. Intuitively, the first term can be thought of as the prediction of the input image **x**_0_, given an iteration step *t*. The second term can be thought of as a vector from **x**_*t*−1_ towards the current iteration step image **x**_*t*_. The third term is random noise. If we substitute in **x**_*t*_ from equation (6.10), we make this intuition explicit,

$$x_{t-1}=\sqrt{{\bar{\alpha }}_{t-1}}\;x_{0}+\sqrt{1-{\bar{\alpha }}_{t-1}-\sigma_{t}^{2}}\;\frac{x_{t}-\sqrt{{\bar{\alpha }}_{t}}\;x_{0}}{\sqrt{1-{\bar{\alpha }}_{t}}}+\sigma_{t}z.$$

If we then set *σ*_*t*_ = 0, we remove the noise dependency and the forward process becomes deterministic,

6.11 $$q_{\text{DDIM}}(x_{t-1}|x_{t},x_{0})=\sqrt{{\bar{\alpha }}_{t-1}}\;x_{0}+\sqrt{1-{\bar{\alpha }}_{t-1}}\;\frac{x_{t}-\sqrt{{\bar{\alpha }}_{t}}\;x_{0}}{\sqrt{1-{\bar{\alpha }}_{t}}}.$$

**Figure 21.**
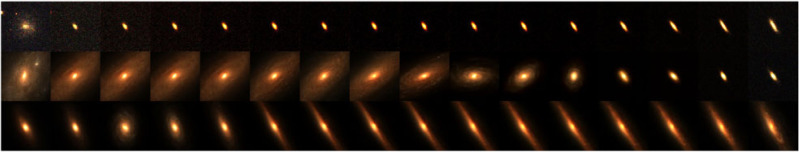
Meaningful latent space interpolation via a DDIM model [13,213]. This property comes ‘for free’ with most other generative models; however, the denoising diffusion probabilistic model [194] requires a tweak to its sampling scheme (equation (6.11)).

This means that DDIMs can deterministically map to and from the latent space, and so inherit all the benefits of this property. For example, two objects sampled from similar latent vectors share high-level properties, latent space arithmetic is possible, and we can perform meaningful interpolation within this space. We demonstrate DDIM latent space interpolation in figure 21.

We can also subsample every *τ* number of steps at inference time, where *τ* is a set of evenly spaced steps between 0 and *T*, the maximum number of steps in the forward process,

6.12 $$q_{\text{DDIM}}(x_{\tau_{i-1}}|x_{\tau_{i}},x_{0})=\sqrt{{\bar{\alpha }}_{t-1}}\;x_{0}+\sqrt{1-{\bar{\alpha }}_{t-1}}\;\frac{x_{\tau_{i}}-\sqrt{{\bar{\alpha }}_{t}}\;x_{0}}{\sqrt{1-{\bar{\alpha }}_{t}}}.$$

As shown in Song *et al.* [213], this results in acceptable generations with a *T*/*τ* inference speed-up.

SBGMs have emerged as a promising alternative to GANs, VAEs and other generative models, showcasing their ability to generate high-quality samples with a level of detail comparable to that of the previous state of the art [201–203]. One of the key advantages of SBGMs is how easy they are to train; they do not inherit any of the instability issues that plague GANs. However, SBGMs do have their share of weaknesses. For instance, the SBGM sampling process is computationally expensive and slow. This is because generating a single sample requires a pass through a learnt Markov chain (figure 20), which can limit their practicality in certain applications. Finally, diffusion models and other SBGMs have not been as extensively explored in the deep learning literature as VAEs and GANs (although this is changing fast!). This leaves their applicability across various domains still under investigation.

## 7. Representation learning

Self-supervised^31^ representation learning has recently exploded in popularity, with a slew of models being developed in rapid succession (e.g. [214–219]). At its core, representation learning attempts to produce semantically meaningful compressed representations (or embeddings) of complex highly dimensional data. Aside from simply being a compression device, these embeddings can also be taken and used in downstream tasks, like clustering, anomaly detection or classification.

In this section, we will describe two approaches to representation learning that are popular within astronomy. The first approach uses contrastive learning as defined by the SimCLR model. The second approach defines and uses a ‘surrogate task’ (such as autoencoding or next-value prediction) to train a deep learning model, and extracts semantically meaningful representations from the subsequent trained network.

### 7.1. Contrastive learning

**Figure 22.**
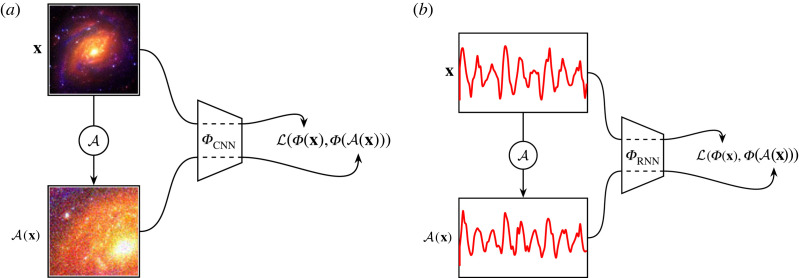
A simple contrastive learning model is applied to both imagery and sequential data. A is an augmentation pipeline. For imagery, A could consist of random crops, noise addition, and colour jitter. For sequential data, A could consist of noise addition, stochastic temporal shifting, and random data deletion. Φ is a function approximator that projects inputs onto an embedding space. Φ is typically a neural network: when processing imagery, Φ could take the form of a CNN, and when processing sequential data Φ could be an RNN. The loss L measures the distance between the embeddings $\Phi (x)=z_{i}$ and $\Phi (A(x))=z_{\;j}$, and we train by attempting to minimize this distance while maximizing the distance between dissimilar samples. (*a*) Possible application to imagery. (*b*) Possible application to sequential data.

Figure 22 describes a simple contrastive learning model similar to SimCLR [214]. This model takes as input a sample **x** from the training set, and augments it to produce A(x). This augmentation is performed in such a way that A(x) shares enough semantically meaningful data with **x** to belong to the same class. In the contrastive learning literature (x,A(x)) is known as a positive pair. This positive pair is passed to a Siamese neural network Φ, which projects the high-dimensional input data onto a lower-dimensional ‘embedding space’. All other training set samples are assumed to belong to a different class to **x**, and so can be combined with **x** to produce ‘negative pairs’. Once we produce some embeddings we need to define a loss that clusters similar samples together, while simultaneously pushing away dissimilar samples. Hadsell *et al.* [220] propose such a loss—the maximum margin contrastive loss

$$L(z_{i},z_{\;j})=\delta_{y_{i}y_{\;j}}\;d(z_{i},z_{\;j})+(1-\delta_{y_{i}y_{\;j}})\max(0,m-d(z_{i},z_{\;j})),$$

where *δ* is the Kronecker delta, **z**_*i*_ and **z**_*j*_ are embedding vectors,^32^ *y*_*i*_ and *y*_*j*_ are the class labels for the embedding vectors, and *m* is the margin. *d* is a ‘distance metric’ (such as for example the L1 loss) that reduces to zero in the case where its inputs are identical. If **z**_*i*_ and **z**_*j*_ are a positive pair, the loss pulls the embeddings closer, and if they are a negative pair the loss pushes the embeddings away from each other. The margin imposes an upper distance bound on dissimilar embeddings.

While useful, the maximum margin contrastive loss does not take into account the embedding space beyond the pair it is attending to in each training step. This limitation ultimately results in a less expressive embedding space. The triplet loss [221] solves this issue by taking into account the broader embedding space and simultaneously attracting a positive pair while repulsing a negative pair with each training step,

7.1 $$L(z_{i},z_{\;j},z_{k})=\max(0,\;d(z_{i},z_{\;j})-d(z_{i},z_{k})+m),$$

where **z**_*k*_ is a sampled from a different class to **z**_*i*_, and **z**_*j*_ is sampled from the same class as **z**_*i*_.

**Figure 23.**
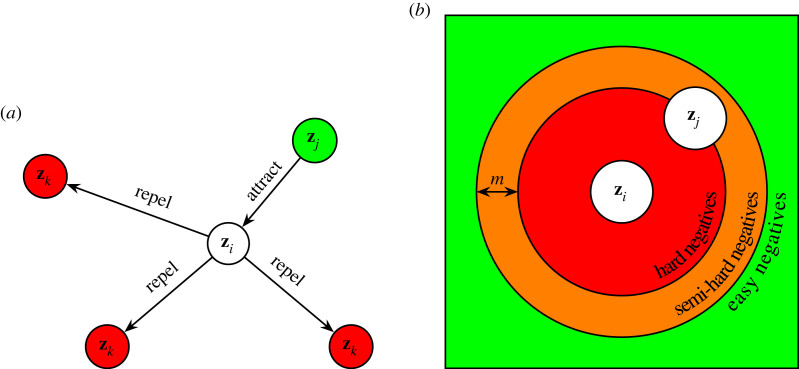
(*a*) The triplet (equation (7.1)) and NT-Xent (equation (7.2)) losses simultaneously incentivize attraction between embeddings sampled from the same class (**z**_*i*_ and **z**_*j*_), and repulsion between embeddings sampled from different classes (**z**_*i*_ and **z**_*k*_). (*b*) Types of negative embeddings. **z**_*i*_ and **z**_*j*_ form a positive embedding pair. If a negative is closer than the current positive it is considered a hard negative, if it lies within the margin it is considered a semi-hard negative, and if it is beyond the margin it is considered an easy negative.

If we study equation (7.1), we see that it is possible to generalize our loss even further, taking into account an arbitrary number of negative samples. The normalized temperature-scaled cross-entropy loss (NT-Xent; [222]) does precisely this,

7.2 $$L(z_{i},z_{\;j})=-\text{log}\left(\frac{\exp(d(z_{i},z_{\;j})/T)}{\sum_{k=1}^{2N}(1-\delta_{ik})\exp(d(z_{i},z_{k})/T)}\right),$$

where **z**_*i*_ and **z**_*j*_ are a positive embedding pair, and **z**_*i*_ and **z**_*k*_ are a negative pair. T is a ‘temperature’ hyperparameter introduced in Chen *et al.* [214] to help the model learn from hard negatives (negatives closer to the anchor than the comparison positive, see figure 23*b*).

### 7.2. Learning representations via a surrogate task

One can also learn representations via a surrogate task. A surrogate task is any task that is unrelated to the network’s final use. However, in the process of learning to perform the surrogate task, the network learns what is important, and what is unimportant about data within the training set. This information can then be extracted in the form of learnt representations. If the surrogate task is general enough, these representations will contain useful semantic information about the items in the dataset, and can then be used for downstream applications.

**Figure 24.**
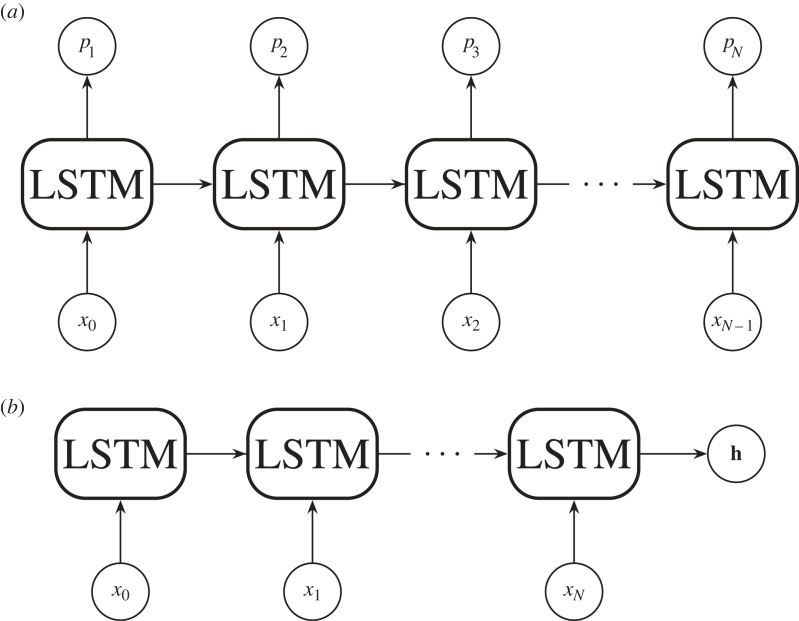
A hypothetical surrogate task for extracting rotation curve representations is shown. {*x*_0_, …, *x*_*N*_} is a set of observations from a galaxy rotation curve, in order of radial distance from the galactic centre. {*p*_1_, …, *p*_*N*_} is the LSTM’s corresponding set of predictions for {*x*_1_, …, *x*_*N*_}. **h** is the LSTM hidden state vector. See figure 11 for more about the internal workings of the LSTM. (*a*) While training we feed in the galaxy rotation curve, and predict the next observation in its sequence. (*b*) While inferring we feed in the full galaxy rotation curve, and extract the LSTM hidden state as a compressed representation embedding of the curve. Otherwise, we ignore whatever output (i.e. {*p*_1_, …, *p*_*N*_}) the LSTM generates.

Let us concretize this process by revisiting an example that we previously discussed in §4.2. Let us imagine we have a large set of galaxy rotation curves that we want to extract embeddings from. We could train an LSTM model (figure 24) on the task of predicting the next item in the rotation curve, with the model only having access to the previous items in the profile. Once the LSTM model is trained on this task, we can feed in a full, new rotation curve and repurpose the final hidden state as a representative embedding. Note that this set-up does not rely on any external labels, only on the rotation curve itself.^33^

We can generate embeddings via an autoencoding task. Again, let us use an astronomical example to specify this and say that we want to extract embeddings from a set of galaxy observations. We could repurpose a variational autoencoder for this, training it as normal as described in §6.1. However, once the model is trained we would discard the decoding part of the network and only consider the encoder. To generate embeddings, we would then simply pass in our galaxy images to the trained encoder. The same process can be carried out by a GAN (§6.2). In the GAN case, we would discard the generator after training and use the discriminator’s penultimate layer outputs as our embeddings.

Supervised networks can also be used to generate embeddings. If a network has been trained in a supervised manner to classify or regress data, it will have learnt some properties about that data that helps it to carry out its task. We can access these learnt representations by taking the outputs from a trained network’s penultimate layer as an embedding.^34^

## 8. Astronomy’s third wave of connectionism

Since its astronomical debut in the mid-2010s [176],^35^ deep generative modelling has become a popular subfield within astronomical connectionism. This popularity is driven by its inherent scalability; the lack of a need for labelled data allows the methods to be repurposed for any dataset that might be at hand. Self-supervised connectionism has been around for longer (i.e. [227]), but again has recently exploded in popularity due to its usefulness in wrangling enormous unlabelled datasets. This section is split into two major parts. We will first outline the history of deep astronomical generative modelling in §8.1, and the history of astronomical representation learning will be discussed in §8.2. Although representation learning is the explicit goal for only the studies described in §8.2, it must be stressed that *representations* *can also be extracted from all the deep generative models described in §8.1.*

### 8.1. Deep astronomical generative modelling

Capturing genuine astronomical data demands accurate knowledge of telescope behaviour, equipment features, environmental factors during observations and data reduction techniques. These complex steps are often tailored to individual observation sets. However, there is an alternative to classical simulation: leveraging examples from a specific survey allows for the development of a data-driven method to simulate not only the astronomical signal but also the inherent characteristics of data. In addition to this, deep learning models trained to replicate astronomical observations are much cheaper to run than classical simulation and so can rapidly generate massive amounts of data; data that can then be used for astronomical pipeline prototyping at scale, aiding the development of new analysis methods, and for dataset augmentation. Data-driven simulation is made possible via the power of deep generative models, and this section describes the history of their use within astronomy.

Seminally, Regier *et al.* [176] proposed the use of a VAE to model galaxy observations. They trained their network on downscaled 69 × 69 crops of galaxies from a SDSS-sampled dataset containing 43 444 galaxies. They trained their network in the same way as described in §6.1, and find that the network is capable of generating galaxies similar to those found in the training set. They also find that their network produces semantically meaningful embeddings, noting that their galaxies are clustered by orientation and morphological type. This same line of enquiry was followed by Ravanbakhsh *et al.* [228], who showed that VAEs could be used to generate galaxies conditionally. Ravanbakhsh *et al.* [228] also pioneered the use of GANs to generate galaxy imagery. Spindler *et al.* [177] used a VAE combined with a Gaussian mixture model prior (see equation (6.2) and accompanying text) to generate and cluster galaxy images into morphological types. While the previous studies in this paragraph used images with relatively small pixel dimensions in their training set, Fussell & Moews [229] and Holzschuh *et al.* [230] demonstrated that GANs are capable of generating large high-fidelity galaxy observations. Fussell & Moews [229] achieved this with a stacked GAN architecture [231], and Holzschuh *et al.* [230] use the related StyleGAN architecture [189] to the same end. Bretonnière *et al.* [12] use a flow-based model^36^ [233,234] to conditionally simulate galaxy observations. They found that their approach could produce more accurate simulations than the previous analytical approach, at the cost of inference time. Relatedly, Smith *et al.* [13] use a diffusion model to generate large high-fidelity galaxies. They trained their network on two datasets comprising galaxies as observed by the Dark Energy Spectroscopic Instrument (DESI, [235]). One, a set of 306 006 galaxies catalogued in the SDSS Data Release 7 [81,236,237], and the other a set of 1962 late-type galaxies, as catalogued in the Photometry and Rotation curve OBservations from Extragalatic Surveys (PROBES, [238]) dataset. PROBES contains well-resolved galaxies that exhibit spiral arms, bars and other features characteristic of late-type galaxies. They found that their model produces galaxies that are both qualitatively and statistically indistinguishable from those in the training set, proving that diffusion models are a competitive alternative to the more established GAN and VAE models for astronomical simulation. From all of these studies, we can conclude that deep generative models can internalize a model capable of physically and morphologically describing galaxies.

Generative models have also been used to simulate astronomical data on larger scales. In a use case tangential to galaxy generation, Smith & Geach [239] show that a Spatial-GAN [240] can simulate arbitrarily wide field surveys. They train on the Hubble eXtreme Deep Field, and find that galaxies ‘detected’ within their model’s synthetic deep fields are statistically indistinguishable from the real thing. Cosmological simulations have also been explored, with Rodriguez *et al.* [241] using a GAN to generate cosmic web simulations at pace, and Mustafa *et al.* [242] generating weak lensing convergence maps at a pace faster than classic simulations. Beyond GANs, Remy *et al.* [243]^37^ trained a SBGM on simulated maps from MassiveNus [245], and found that their model was capable of replicating these maps. They also demonstrated that their model was capable of producing a likely spread in the posterior predictions. Finally, they demonstrate that a SBGM is capable of predicting the mass map of the real Hubble Cosmic Evolution Survey (COSMOS) field [246].

The image domain translation abilities of GANs in a Pix2Pix-like formulation ([184], also see figure 19*b*) is particularly useful in astronomy. Schawinski *et al.* [247] demonstrated this use first by training a Pix2Pix-like model to denoise astronomical data. They trained their network on 4550 galaxies sampled from SDSS. The galaxies were convolved to increase the seeing, and speckle noise was added. The GAN was tasked with reversing this process. They found that their method outperformed both blind deconvolution, and Lucy–Richardson deconvolution. Generative models are also capable of separating sources, as Stark *et al.* [248] demonstrate by using a Pix2Pix model to deblend a quasar’s point source emission from the extended light of its host galaxy. Reiman & Göhre [249] use a similar model to Stark *et al.* [248] to deblend overlapping galaxies.

At the time of writing, there are only three examples of score-based (or diffusion) modelling in the astronomy literature [13,243,244].^38^ It is surprising that these studies are the only examples of score-based modelling in astronomy, as SBGMs produce generations that rival that of state-of-the-art GAN models, without drawbacks present in other models (like blurring in the case of VAEs, or mode collapse and training instability in the case of GANs). SBGMs also have some natural uses in astronomical data pipelines. For example, an implementation similar to Sasaki *et al.* [206] could be used for survey-to-survey photometry translation similarly to Buncher *et al.* [254]. The source image separation model described in Jayaram & Thickstun [207] has the obvious application as an astronomical object deblender (i.e. [248,249,255]). To summarize, SBGMs are ripe for exploitation by the astronomical community, and we hope to see much interest in this area in the coming years.

### 8.2. Self-supervised astronomical representation learning

In 1993, Serra-Ricart *et al.* [227] proposed using an autoencoder to learn embeddings for stars as observed by the Two Micron Galactic Survey [256]. They first proved that their autoencoder model worked better than principal component analysis (PCA) on the toy problem of separating Gaussian distributions, and they then showed that their model also outperformed the classic PCA method on real data. More than 20 years later, Graff *et al.* [257]^39^ showed that autoencoders are also capable of capturing the properties of galaxies as described in the Mapping Dark Matter Challenge [258] by demonstrating that embeddings extracted from their autoencoder were beneficial for computing the ellipticities of their galaxies as a downstream task. We are not limited to imagery; Yang & Li [259] show that an autoencoder can learn representations that can then be used to train a neural network for the downstream task of estimating stars’ atmospheric parameters, and Tsang & Schultz [260] demonstrate that an autoencoder can generate embeddings that can then be used to classify variable star light curves. From these studies we must conclude that neural networks trained via a surrogate task are capable of learning semantically meaningful embeddings across astronomical domains.

Very recently, there has been work applying self-supervised contrastive learning models to galaxy image clustering. Hayat *et al.* [11] trained SimCLR [214] on multi-band galaxy photometry from the SDSS [81]. They show that the resulting embeddings capture useful information by directly using them in a training set for a galaxy morphology classification model, and a redshift estimation model. Similarly, Sarmiento *et al.* [261] trained SimCLR on integral field spectroscopy data captured from galaxies in the Mapping Nearby Galaxies at Apache Point Observatory survey (MaNGA, [262]). Again, they find that SimCLR produces semantically meaningful embeddings. Slijepcevic *et al.* [263] demonstrate that the ‘Bootstrap Your Own Latent’ (BYOL, [216])^40^ contrastive learning model is capable of learning semantically meaningful representations of radio galaxies. Their model is trained on 100 000 Radio Galaxy Zoo galaxies, and inference is run on the 1256 galaxy strong Mirabest dataset [264]. They find that embeddings derived from their model are semantically meaningful, suggesting that self-supervised methods are transferable between disparate surveys. These studies show that contrastive learning is applicable to imagery; further study will be required to demonstrate its effectiveness with other types of astronomical data, such as time-series and volumetric data.

## 9. Foundation models: a fourth astroconnectionist wave?

This review has shown thus far that deep learning has found wide use in astronomy, a use predicated on the availability of enormous amounts of computational power and data. This section looks to the future and predicts an outcome if astronomy continues to follow in the footsteps of other applied deep learning fields. In short, we predict and argue that astronomical connectionism will probably see the removal of expertly crafted deep learning models, to be replaced with an all encompassing ‘foundation’ model. In §9.1, we explore what foundation models are, and their context within deep learning. Section 9.2 then contextualizes these models within astronomy, and suggests actions we can take as a community to realize an astronomical foundation model. Finally, §9.3 demonstrates as a thought experiment a state-of-the-art use case for an astronomical foundation model and explores other theoretical and practical uses and implications within (and beyond) astronomy.

### 9.1. Foundation models

Since its inception, connectionism has followed a path of greater compute and greater generality [91,92]. In that time, human-crafted biases have fallen by the wayside, to be replaced with models and techniques that learn directly from data. Sutton [91] exemplifies this process via the field of speech recognition:

In speech recognition, there was an early competition, sponsored by DARPA [Defense Advanced Research Projects Agency], in the 1970s. Entrants included a host of special methods that took advantage of human knowledge—knowledge of words, of phonemes, of the human vocal tract, etc. On the other side were newer methods that were more statistical in nature and did much more computation, based on hidden Markov models (HMMs). Again, the statistical methods won out over the human-knowledge-based methods. This led to a major change in all of natural language processing, gradually over decades, where statistics and computation came to dominate the field. The recent rise of deep learning in speech recognition is the most recent step in this consistent direction. Deep learning methods rely even less on human knowledge, and use even more computation, together with learning on huge training sets, to produce dramatically better speech recognition systems. As in [computer Go and computer chess], researchers always tried to make systems that worked the way the researchers thought their own minds worked—they tried to put that knowledge in their systems—but it proved ultimately counterproductive, and a colossal waste of researcher’s time, when, through Moore’s Law, massive computation became available and a means was found to put it to good use.

We are seeing this principle play out once again through a new paradigm shift in deep learning, where even the underlying neural network architecture does not matter. Previously, neural networks were adapted for a specific domain via inductive biases injected by researchers, such as convolutions for computer vision, and recurrence for language processing. Now we are seeing transformer networks (see §4.4 and [117]) competing^41^ in all deep learning domains applied or otherwise: from language processing [17,123]^42^ to computer vision [18,168] to graph learning [267] to protein folding [16] to astronomy [169,170,172]. The transformer’s versatility allows us to take a model trained on one task and apply it to a similar yet different task, a process known as transfer learning. For example, we could train a model on the ‘surrogate’ task of predicting the next word in a sequence, and then apply that model to a similar yet different task of predicting the answer to a geography question. In this example, the first model is known as a ‘foundation’ model, and the downstream model is derived from it. This set-up brings with it some useful advantages. For example, if the foundation model is improved, all downstream tasks also see improvement. Therefore, the need for only one model allows researchers to pool their efforts in a way not possible when resources are split between many projects.

To train a foundation model, we first need to define a surrogate task. As labelled datasets are expensive, and raw data are relatively cheap, the easiest and most scalable way to do this is via self-supervised learning.^43^ Self-supervised learning does not require a human to provide a labelled dataset for training. Instead, the supervisory signal is generated automatically from the raw data. For example, in the context of astronomy this task could be predicting a masked value in a variable star’s light curve [169]. Another task could be using an autoencoder (§6.1) to replicate a galaxy observation [177]. A further task could be training within a self-supervised framework, like contrastive learning (§7.1). The important thing about self-supervised learning is that it does not require annotated data. This means that we can leverage vast reserves of raw data (such as textbooks, scraped Internet text, raw imagery, etc.).

**Figure 25.**
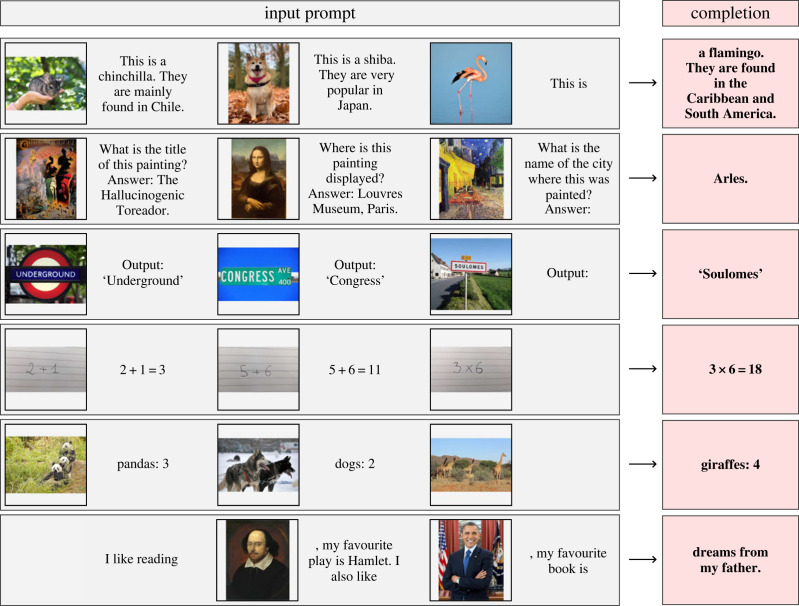
Flamingo is a foundation model that is capable of understanding images within the context of natural language. Here we see some examples of Flamingo’s emergent abilities. This figure is adapted from fig. 1 in Alayrac *et al.* [268].

Very large models trained on vast amounts of data demonstrate surprising emergent behaviour. For instance, GPT-3 [17] is a 175 billion (B) parameter model that can be ‘prompted’ to perform a novel task (see figure 25 for more on prompting foundation models). This ability was not shown at all in GPT-3’s older, smaller 1.5B parameter sibling [122]. Furthermore, a meta-study described in Wei *et al.* [269] found that larger models suddenly ‘unlock’ abilities such as arithmetic, translation and understanding of figures of speech once they reach a certain scale. These findings suggest that architectural changes are not required beyond scaling to perform many tasks in natural language processing [92,270]. In figure 25, we see some results from Alayrac *et al.* [268], a model comprising an LLM, and an image encoder. In this figure, we can see that the model is capable of arithmetic, reading, counting and has a broad knowledge (albeit not ‘understanding’) of art, geography and zoology,^44^ and literature. This model comprises a ResNet variant [119,272] to encode imagery, and the Chinchilla LLM [273] to encode and generate text. Chinchilla (and therefore Flamingo) was trained with the surrogate task of predicting the next word in a text sequence, and so none of the emergent properties stated above were explicitly optimized for.

In the next subsection, we will state and explain the need for an astronomical foundation model,^45^ not only for astronomy’s sake, but also for the sake of openness in deep learning research.

### 9.2. Scaling laws and data moats

Hoffmann *et al.* [273] suggested an update to the foundation model scaling law first proposed in Kaplan *et al.* [275]. Their scaling law equation relates the size of a neural network model and the training dataset size to the minimum achievable loss. Mathematically, the equation is

9.1 $$L_{\min}(N,D)=\underset{\text{parameter term}}{\underbrace{\frac{A}{N^{\alpha }}}}+\underset{\text{data term}}{\underbrace{\frac{B}{D^{\beta }}}}+\underset{\text{dataset entropy}}{\underbrace{E}},$$

where *E* is a constant that represents the lowest possible loss, given a particular training dataset. *N* is the number of trainable parameters within the neural network, and *D* is the size of the dataset in tokens (see §4.4 for more about tokenization). We can see that when we have an infinitely large model trained on an infinitely large dataset (i.e. *N* = *D* = ∞), the only term remaining is the ‘dataset entropy’ constant, *E*. We can therefore only reduce the loss by increasing the size of our model, or the size of our training set.

After fitting equation (9.1), Hoffmann *et al.* [273] find

$$L_{\min}(N,D)=\frac{406.4}{N^{0.34}}+\frac{410.7}{D^{0.28}}+1.69.$$

**Figure 26.**
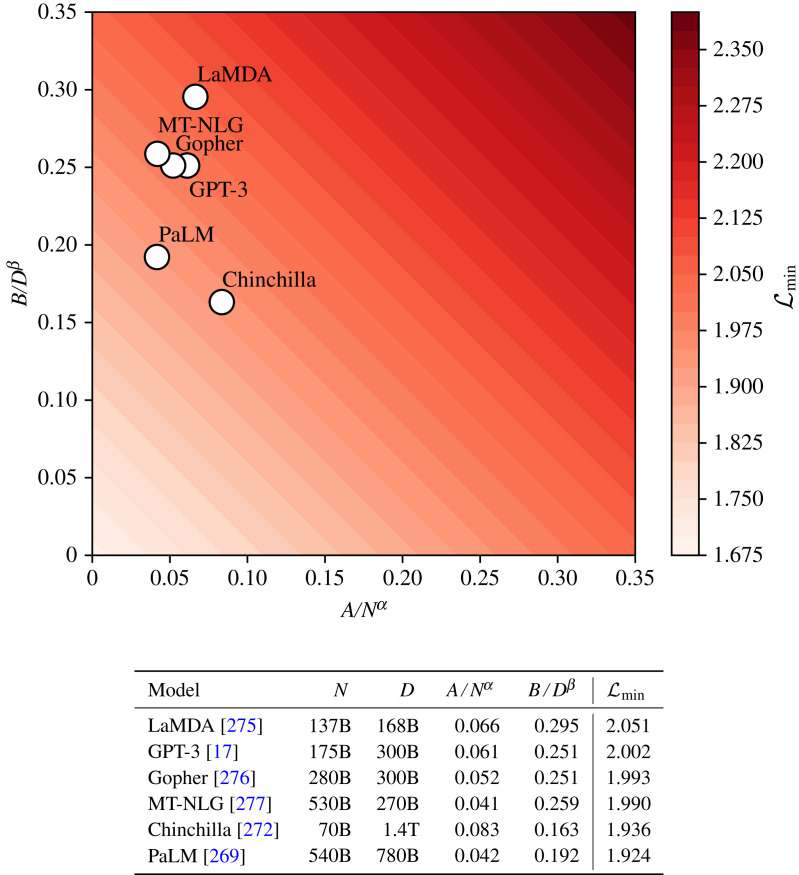
A comparison between the minimum losses of a selection of foundation models. The table above shows the number of parameters in a model (*N*), the number of tokens within that model’s training set (*D*), and their corresponding calculated emergent terms from equation (9.1). Here we use Hoffmann *et al.* [273] to source values for *A*, *α*, *B* and *β*. The minimum loss for each model accordingto Hoffmann *et al.* [273] is shown as $L_{\min}$. The contour plot shows the emergent parameters $B/D^{\beta }$ and $A/N^{\alpha }$ plotted against each other for our models. The closer the models’ scatterpoints are to the bottom left, the lower their minimum loss value.

If we then plug in *N* and *D* for a selection of real foundation models we arrive at figure 26. We can see in figure 26 that the model size term for real foundation models is far lower than the dataset size term. This means that an increase in dataset size has the potential to reduce the minimum loss by a far larger amount than a larger model would. Therefore, an obvious next step to improve these foundation models further is by increasing their dataset size.

The largest dataset (MassiveText-English; [273]) in the comparison shown in figure 26 amounts to 1.4 trillion (T) tokens. However, this dataset is proprietary, being only available to researchers employed by Google. The largest public text dataset available at the time of writing is The Pile [279], with a total size of approximately 260B tokens. We could increase the size of these datasets by indefinitely scraping text data from the surface web, but these data tend to be of low quality. Also, we have already exhausted some important high-quality data reserves, like fundamental research papers, and open source code [280]. We also have to ask ourselves: what happens when generative models start to create data en masse, and dump it indiscriminately onto the Internet? If a significant proportion of text in a dataset scraped from the Internet is generated via an LLM, training on it will cause unforeseen issues and may ultimately result in a model with worse performance. We must therefore ensure that the data are not generated by a deep generative model. In addition to all this, the academy and the public at large will never have access to the vast reserves of data contained in the deep web administered by ByteDance, Google, Meta, Microsoft and other tech giants. For all these reasons, we will need to think outside the box if we want to mine new high-quality data.

Enter the multi-modal foundation model. Reed *et al.* [124]^46^ demonstrated that a large transformer neural network is capable of learning many tasks, from playing Atari, to captioning images, to chatting, to operating a real robot arm. The model shares weights across all tasks, and decides at inference time from context which task to predict. Importantly, Reed *et al.* [124] find that their model follows the same scaling laws as other foundation models, and so multi-modal foundation models have the same hunger for data that we see in figure 26. Even more astonishingly, Aghajanyan *et al.* [282] find that a foundation model trained on concatenated independent datasets significantly outperforms separately trained unimodal models once the neural networks reach a certain scale. We can therefore augment our text datasets with high-quality, publicly available astronomical data.

The Vera Rubin Observatory’s 189 16 megapixel CCDs will observe 1000 science frames per night while conducting the Legacy Survey of Space and Time (LSST) [283]. This amounts to 3 × 10^12^ pixels per night, or approximately 12B tokens a night if we use the same tokenizing scheme as Dosovitskiy *et al.*’s vision transformer [18]. After only 1 year of observing, the LSST will have produced 4.4T tokens of raw data, larger than even the MassiveText-English dataset.^47^ These data, and other astronomical data like it, could be compiled into a very large open dataset similar to EleutherAI’s Pile [279]. This dataset would provide a way for academics employed outside of Big Tech to train and research very large foundation models. Compiling a dataset like this would be difficult for a single relatively under-resourced research group, but it could be accomplished via bazaar style open development [284]. We have already seen this development model succeed in large open source projects, the most famous of which is the Linux kernel. This development model has also been shown to work within the field of deep learning by EleutherAI (e.g. [279,285,286]), and with HuggingFace’s BigScience initiative [287]. Once compiled, we must ensure that progress is kept in the open, and that the data are not simply absorbed into proprietary datasets—to do this we must give our dataset a strong (viral) copyleft style licence.

Once the dataset is compiled all we need for training are some self-supervised surrogate tasks for our ‘astrofoundation’ model to attempt. These tasks could include predicting the next observation in a variable star’s time sequence, predicting the low surface brightness profile of a galaxy, predicting a galaxy’s morphological parameters or simply generating the next crop in a sequence of observations.^48^ As we will explore in the next subsection, these surrogate tasks do not need to be at all related to the downstream tasks we will eventually use our model for. Once trained, our astrofoundation model will inherit all the interesting properties that LLMs enjoy, such as few- to zero-shot generation and other emergent behaviours.

### 9.3. The practical implications and uses of an astrofoundation model

This section explores the wider implications of a hypothetical astrofoundation model (§9.3.1), as well as some practical astronomical uses (§9.3.2). In §9.3.3, we highlight one possible downstream task that would be useful in astronomy; a conditional generative model for astronomical simulation.

#### 9.3.1. Democratizing foundation models

**Figure 27.**
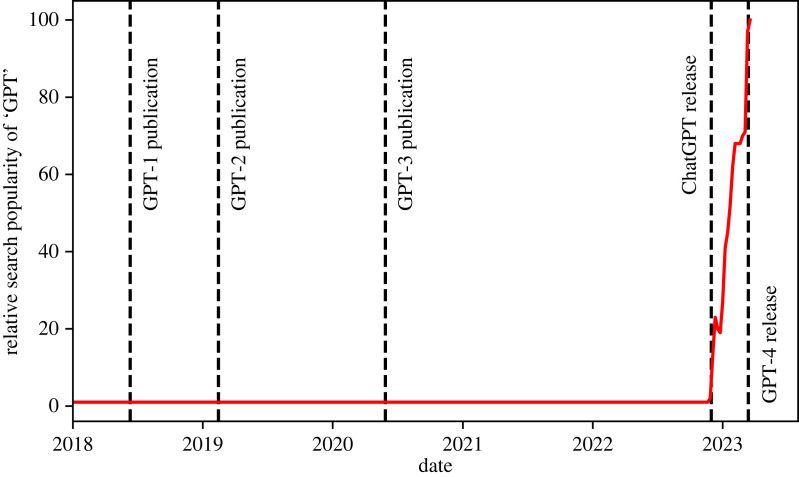
Here we show the relative Google search popularity for the term ‘GPT’. We can see a huge increase in searches for GPT when the ChatGPT model was launched for public use (and surprisingly little increase in searches when the GPT-1, GPT-2 and GPT-3 papers were released!) [17,26,122,290]. These data are taken from Google Trends (https://trends.google.co.uk/).

The spring of 2023^49^ has brought with it a shift in the global zeitgeist’s attention towards foundation models in general, and the GPT family of large language models in particular. Leading the charge is OpenAI’s ChatGPT, whose release has become a very public advertisement of the abilities that large language models possess (figure 27). While impactful, we note that ChatGPT is ‘just’ a web interface wrapper for versions of GPT-3 and GPT-4 that have been fine-tuned using human feedback [291,292]. ChatGPT’s popularity therefore suggests that there is a lot of latent general interest in deep learning and foundation models, and that this interest can be realized through a convincing public demonstration. Fully open development and dissemination of these models is perhaps the most public demonstration there is. And we have indeed seen that the release of open source foundation models leads to an explosion of innovation and interest.^50^ One particular example is the release and impact of the ‘large language model [from] Meta AI’ (LLaMA; [293]). The LLaMAs are a collection of open source LLMs, and the largest LLaMA has a comparable performance to GPT-3. Since LLaMA’s release, an entire ecosystem of projects have spun up that use the model in innovative and interesting ways (e.g. [294–297]). A similar story occurred in 2022 when StabilityAI released an open text-to-image diffusion model based on latent diffusion [94]. The following flurry of activity far outstripped the progress OpenAI made with their competing closed source DALL-E 2 model [203,298]. We believe that a similar explosion of innovation to that seen with the release of the LLaMA and Stable Diffusion models would lay in store for astronomy if an open astronomical foundation model is developed and marketed effectively.

**Figure 28.**
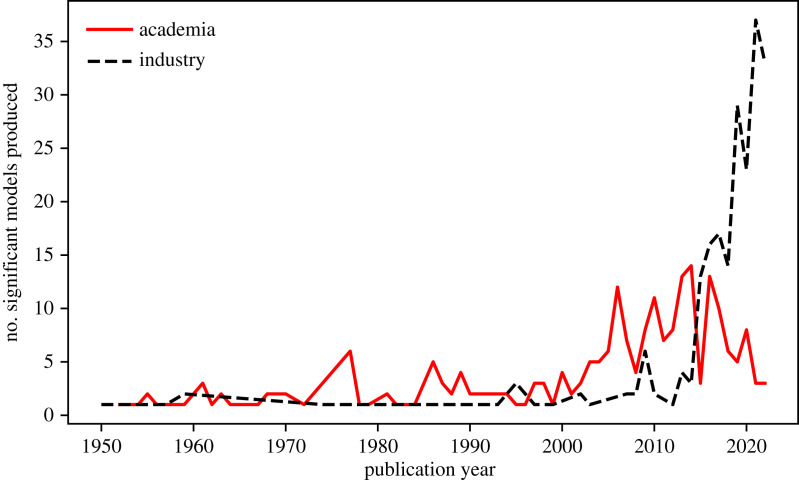
Here we show the number of highly cited, state-of-the-art or historically significant works produced per year within academia and industry. These data are taken from Sevilla *et al.* [299].

In mid-March GPT-4 was released [26]. Its accompanying ‘Technical Report’ contains no detail on the model’s architecture, training set size, or training routine.^51^ The unashamed release of a closed model is quite a worrying development for a field that has historically been built on open source and open research. Of most concern is industrial actors within this space closing up shop as a reaction to the open/closed model prisoner’s dilemma set by OpenAI. As figure 28 shows, industry has produced the lion’s share of impactful deep learning models since the mid-2010s; if future developments are kept hidden due to commercial pressure we will see a concentration of talent and innovation locked away behind industry’s closed doors. Furthermore, the latest developments in foundation modelling have the potential to significantly impact the global economy and workforce through pervasive automation [173,300]. As automation increases, the concentration of power, expertise and economic clout within large industrial actors will weaken the economic bargaining position of those that do not have access to these technologies. This could result in a societal equilibrium where fewer and fewer people have access to economic and social opportunity. This is an equilibrium that Brynjolfsson [301] memetically dubs the ‘Turing Trap’:

A fully automated economy could, in principle, be structured to redistribute the benefits from production widely, even to those who are no longer strictly necessary for value creation. However, the beneficiaries would be in a weak bargaining position to prevent a change in the distribution that left them with little or nothing. They would depend precariously on the decisions of those in control of the technology. This opens the door to increased concentration of wealth and power.

To avoid this trap, we must collectively work towards making foundation models—and by proxy the latest fruits of automation—available to all. A copyleft foundation model trained on a copyleft dataset (such as our hypothetical astronomical foundation model) would go some way towards reducing the growing technological inequality between Big Tech and wider society.

With the above discussion in mind, we would like to revisit our brief analysis in §9.2 and restate and emphasize the pressing need for an independent, verifiable, completely open and strong copyleft licensed alternative to the closed foundation models controlled by OpenAI, Microsoft, Anthropic, Google and other Big Tech conglomerates. While expensive, the compute is fairly easy to source—the paramount issue is that foundation models require a huge amount of data to train them effectively. These models are usually trained via a large amount of high-quality publicly unavailable textual data that is locked within the deep web. Fortunately, however, §9.2 shows that a large amount of useful multi-modal data can be easily sourced from astronomical observations. We can therefore conclude this subsection on a positive note—astronomy is ideally poised to play an outsized role in the democratization of foundation models.

#### 9.3.2. Possible astronomical use cases

In this subsection, we outline some possible exciting astronomical uses for our astrofoundation model. Before we dive in, we must state that here we only skim the surface of this technology’s potential, and we hope that—as evidenced by the LLaMA and Stable Diffusion ecosystems (§9.3.1)—there will be many more use cases that we have not discussed here that would emerge from community involvement. We divide this subsection into two parts. The first part talks about how a foundation model could aid outreach, citizen science and cross-disciplinary collaboration, and the second part discusses how the model could aid astronomical research.

##### 9.3.2.1. Collaboration, citizen science and outreach

By providing a common platform for generating simulations and analysing data, a neural network-based astrofoundation model would ease and facilitate collaboration between researchers in previously disparate fields. In addition to this, any improvement in the underlying technology could easily be integrated into field-specific (or field-agnostic) foundation models that could be used for tasks that previously needed years of specialist training to operate. One example specific to astronomy is astronomical simulation. A physically aware astrofoundation model could be used to simulate and interrogate simulated astronomical events in much the same way that classical simulations do now [20–22]. Section 9.3.3 describes in detail one framework that could facilitate such a model.

The multi-modal training of neural networks lets us make connections between data modes that would be impossible or difficult with current methods. As just one example, let us consider citizen science. In a citizen science project like Galaxy Zoo [132], citizen scientists are asked to label astronomical objects with quantitative labels. This can be an unintuitive process for someone untrained in astronomy. An astronomical foundation model that has an awareness of natural language would allow participants to describe astronomical objects using their own words. This would reduce the need for specialized training and therefore would increase the accessibility of these projects. One could imagine a new Galaxy Zoo-like project where citizen scientists provide natural language descriptions of galaxy morphologies. The foundation model could then process and analyse these descriptions, which would eventually contribute to a more comprehensive understanding of galaxy evolution.^52^

A foundation model with astronomical knowledge could be used to develop chatbots capable of engaging students, educators and the general public in conversations about astronomy. These chatbots could answer questions, provide explanations, or even suggest personalized learning resources based on the user’s interests and prior knowledge. This would widen and democratize access to astronomical knowledge, and this easy access to astronomical knowledge could enthuse and help to recruit the next generation of astronomers. Foundation models can already act as tutors, and commercial actors are currently working in this space; the most notable examples being ‘Duolingo Max’ which provides users a personalized chatbot for foreign language learning, and Khan Academy’s ‘Khanmigo’ which provides students a personal tutor for their courses. Both Duolingo Max and Khanmigo are paid offerings powered by OpenAI’s GPT-4 API [26], and so an open astronomical foundation model would provide wider access than a closed GPT-*N* model that has been prompted to become astronomically aware.

##### 9.3.2.2. Augmenting research

While the foundation model is necessarily trained on existing data, its ability to identify patterns and relationships within the data can lead to new knowledge discovery, and a more efficient way to process data that previously was difficult or time consuming. As discussed previously in §§6–8, an astroconnectionist could use the foundation model to generate embeddings for a set of astronomical objects. Like we discussed in §§6–8, these embeddings could be used for downstream astronomical tasks, or could be placed into visualization pipelines like the t-distributed stochastic neighbour embedding method [303,304]. Since the astronomical foundation model would be multi-modal, a researcher could combine the embeddings of multiple datasets generated from entirely different instruments, giving them a birds-eye view of their data that would currently be difficult to achieve. We can also use the foundation model’s emergent abilities to our advantage; as shown in figure 25 we could use few-shot learning and prompt the trained model with a few example pairs of inputs. For instance, we could use pairs of input galaxy observations and corresponding output surface brightness profiles [167]. If the astronomical foundation model is a few-shot learner (and is aware of a similar input–output pairing within its training data), it would identify that the researcher wants to calculate the surface brightness profiles of new galaxies. The researcher would then use the prompted model as a surface brightness profile extractor, sidestepping the need for a specialized analytical or deep learning model for such a task. This process is not limited to this example—it would work for any input–output pair within a mode that the foundation model is aware of. Even better, this process would require no retraining of the foundation model, it would only require the few-shot prompt at inference time.

Autonomous agents are no longer science fiction; task-driven autonomous agents powered by the simulacra of a foundation model are capable of solving very general tasks when given only a high-level prompt by a human operator [305,306]. One could therefore imagine a semi-automated research pipeline, where an autonomous agent with astronomical knowledge is given access to a set of astronomical data through an API. The agent would be prompted with a high-level research goal (such as ‘find something interesting and surprising within this dataset’), and would then take steps to achieve this task. These steps could include querying research papers for a literature review, searching a large multi-modal astronomical dataset to find data that supports a theory, evoking and discussing its findings with additional simulacra, or spinning up simulations to test a hypothesis [307]. While the agent operates in the background, the human researcher would be able to provide high-level interpretation of the results, and would be a steady hand providing guidance and refinement of a more general research direction. In this way, an astronomical foundation model would provide the tools to make all astronomers the principal investigator of their own powerful ‘AI lab’.

#### 9.3.3. A new class of simulation

We would like to end this subsection with a tangible application of our hypothetical astrofoundation model; a conditional generative model for astronomical simulation in the spirit of recent work on text-to-image modelling (i.e. [94,308]). If we train an unconditional generative model, we cannot control its output at inference time. This is an issue if we want to generate specific classes of observations to train models for downstream tasks, such as redshift estimation, or galaxy-type classification. To achieve a model capable of generating specific classes, one could simply train a conditional generative model of the form

9.2 $$G_{\phi }(\hat{x}|z,y),$$

where $\hat{x}$ is a generated image, **z** is some noise that acts to capture all detail not encoded in **y**, and **y** is a conditioning vector. As an example, **y** could contain a galaxy’s redshift or morphological type. However, this means that we must be very specific when choosing **y**. Multi-modal modelling provides us the means to sidestep this fundamental issue, and lets us play with fuzzy inputs.

**Figure 29.**
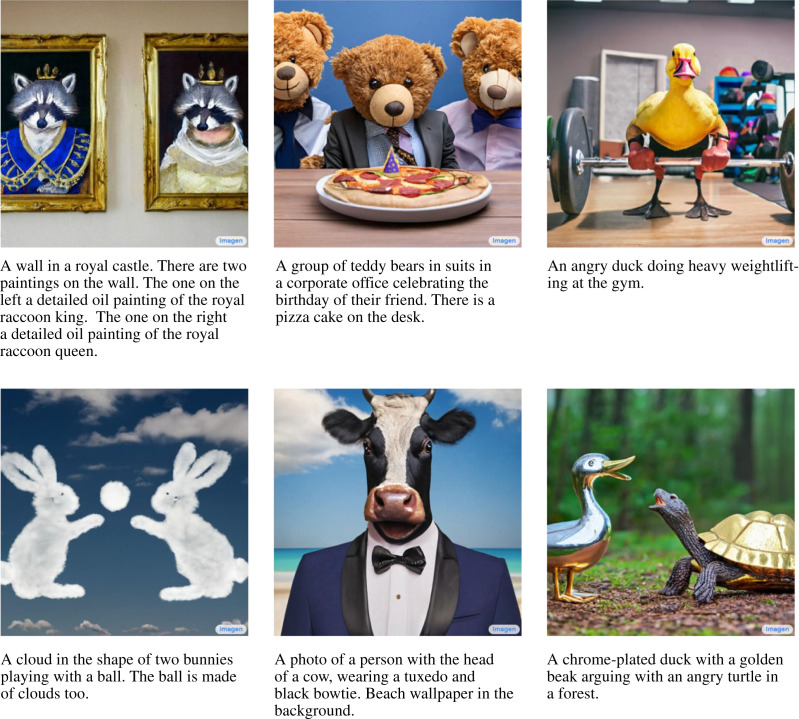
Select 1024 × 1024 Imagen samples generated from text inputs. Below each image is its corresponding conditioning text. Figure adapted from fig. A.2 in [308].

**Figure 30.**
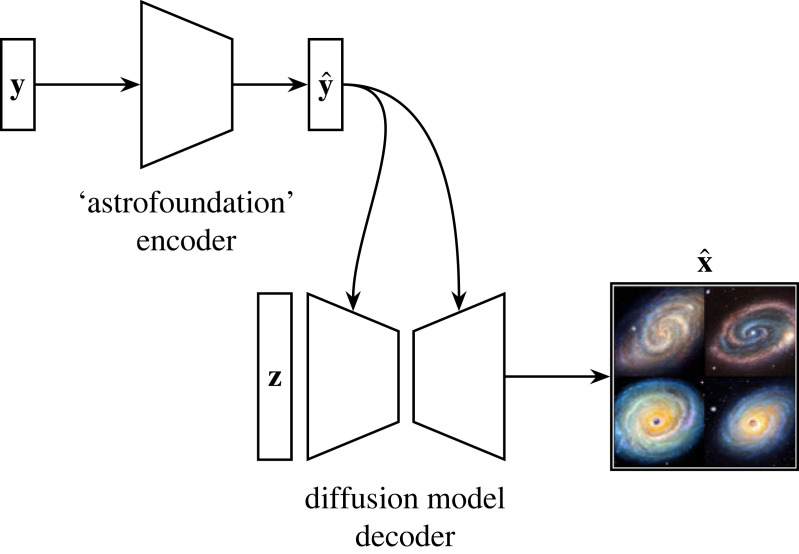
An Imagen-like model uses a frozen foundation model to encode text, and then uses that encoding to condition a cascaded diffusion model of the form $G_{\phi }(\hat{x}|z,\hat{y})$ [308,309]. Here we see one possible realization of this type of model in astronomy. **y** is some kind of descriptive vector that can be paired with a ground truth image. For example, **y** could be the surface brightness profile of a galaxy, or the summary statistics of a variable star light curve, or some cosmological parameters. In general, **y** could be any vector that the astrofoundation model understands. $\hat{y}$ is **y**’s projected latent space equivalent. Since we do not need to train the foundation model here, training cost is far lower than for an equivalent end-to-end trained model.

As a thought experiment, let us consider Google’s recent ‘Imagen’ model,^53^ and imagine how it could be repurposed for an astronomical use case (figures 29 and 30, [308]). Imagen is a combination of a frozen LLM (specifically T5-XXL; [310]) and a cascaded diffusion model ([309], also see §6.3). The LLM acts as a language encoder, and then passes its generated latent space representations onto the diffusion model as a conditioning vector. If we were to replace the frozen LLM with an ‘astrofoundation’ model (see §9.1 and 9.2), we could leverage astronomy’s fundamentally multi-modal nature. For example, if our astrofoundation model were trained to understand the Galaxy Zoo 2 (GZ2) morphological classifications [311], we could take the GZ2 descriptors as **y** and their corresponding galaxy pair as **x** and train on those.

Once trained, our astronomical Imagen model could generate synthetic galaxies that resemble the real galaxy observations that it was trained on. However, unlike an unconditional astronomical simulator, this model would be capable of generating galaxies that specifically resemble a real galaxy that shares the conditioning set of GZ2 parameters!

Unlike the conditional model described by equation (9.2), an astrofoundation-type model allows us to be creative with the conditioning vector. For example, we could run the model in reverse to generate representations that refer to a very specific astronomical object, and then generate many more objects of that ‘class’ with injected features like satellite occlusion, a specific instrument response function, a specific redshift, etc. (see work on ‘textual inversion’ by Gal *et al*. [312]). These simulations would enable researchers to create tailored datasets for various research purposes, such as studying particular galaxy types, morphologies or cosmological phenomena. We could even create a ‘Galaxy Zoo’ type dataset that asks citizen scientists to describe galaxy morphology via natural language (§9.3.2). This is possible since the encoding foundation model does not fundamentally care about which form the caption takes. This approach would cut down on citizen scientist training cost due to natural language’s inherent intuitiveness. Furthermore, as inference-time generation is relatively cheap, a model like the one described in this section would allow astronomers to explore and test hypotheses and scenarios more rapidly than they could if they used a classical simulation.

## 10. Connectionism’s caveats

Thus far in this review we have been very optimistic about astronomical connectionism’s potential. However, this does not mean that connectionism is without its pitfalls. Section 10.1 outlines some practical downsides of astronomical connectionism, and discusses how a practitioner can mitigate them. Owing to its importance, we dedicate §10.2 to the discussion of climate change and carbon emissions, and illustrate connectionism’s impact with a case study on the carbon emissions of modern large language and foundation models.

### 10.1. Possible practical pitfalls

As illustrated in figure 26, deep learning has an insatiable hunger for data. Acquiring and labelling data for the training of deep learning models can be extraordinarily expensive and time-consuming. The savvy astroconnectionist could mitigate this problem through self-supervised or generative learning that does not require labelled data, and then repurposing learnt embeddings for more specialized downstream tasks^54^ (see §§6–9). Related to this, rare or entirely unexpected astronomical events and phenomena^55^ are by definition poorly sampled within any training data, and so a deep learning model will have difficulty generalizing and internalizing these events. One solution is using an anomaly detection method to find these rare phenomena. We direct the reader to Pang *et al.* [315] for an excellent recent review of anomaly detection techniques.

Very large deep learning models can be expensive to train and run inference with. Some astronomical applications, such as detecting transient events, require real-time processing of large volumes of data. The computational complexity of deep learning models can pose challenges for their deployment in these time-sensitive scenarios. In that case, it may be preferable to employ a fast, simple, classical technique or to use a smaller deep learning model.

Astronomical data can be observed via a variety of different instruments (or simulations), and the final output data can be processed by any number of post-processing pipelines. These pipelines each have their own characteristics, idiosyncrasies and foibles, and so can appear very different when propagated through a deep neural network. Also, the distribution of known celestial objects within a survey may be influenced by observational biases or historical interests, and so careful inspection of datasets is required to ensure that they are representative for the desired use case. In addition to care, an astroconnectionist might employ domain adaptation techniques to ensure that their datasets are representative for their downstream tasks [316]. Finally, as we explored in §9, it may even be enough to simply train a very large deep learning model on a collection of datasets [282], but this approach is currently out of reach for the average researcher.

The perennial criticism of deep learning is—of course—interpretability. As deep learning models are highly parametrized it is difficult to understand why they arrive at a certain behaviour or decision. There are many ways to sidestep this issue, and this paragraph will briefly outline some developments in this direction that might be of use to a practitioner. Perhaps the gold standard for interpretability is a neural network walking the user through its ‘thought’ process step-by-step with natural language, as a human would do. Large language foundation models can do this, and this ability comes ‘for free’ with a sufficiently large model and dataset [317]. Unfortunately, however, no such foundation model currently exists that also has a deep knowledge of astronomy (§9) so we must be a little more creative. Attentional mapping can be used to show which features the deep learning model are attending to when producing an output, and this attentional mapping can be depicted as a heat map over our data. Attentional mapping can be generated in several ways; for example, we could use a mechanism like we discussed in §4.4 to highlight the most useful parts of an input datum for the model to predict or generate its output. One can also use class activation mapping [231] to trace the outputs of a fully convolutional neural network back to its inputs to see which parts of an input image are used in a prediction. Occlusion mapping (and other perturbation techniques) can be used to visualize attention for all architectures. Occlusion maps require us to occlude parts of an input datum and in turn allow us observe how that affects the output prediction [137]. We can also apply certain statistical methods to deep learning models to gain an insight into their inner workings. Stochastic neural networks trained within the Bayesian paradigm (or ‘Bayesian neural networks’) can be used to estimate the uncertainty in neural network predictions [318]. One does not need to have prior knowledge of the dataset when training a Bayesian neural network; neural networks can make use of approximate Bayesian computation techniques like likelihood-free inference to estimate the posterior [319]. Besides these methods, many other deep interpretability pipelines are in use—far more than we have space to go over here—and so we highly recommend Ras *et al.* [320] for a general and extensive overview of the field of explainable deep learning.

### 10.2. Connectionism’s carbon crisis

The training of deep learning models in general requires a considerable amount of energy, and it is only natural that the training of ultra-large foundation models significantly ups the ante. In this section, we illustrate connectionism’s hunger for energy by estimating the total carbon footprint created in the training of the GPT-3^56^ and PaLM foundation models [17,270].

Let us start with the eminent GPT-3 model. Unfortunately, the total energy cost is not stated in Brown *et al.* [17] but we can make a ballpark estimate using information from that work. GPT-3 was trained on a high-performance computing cluster containing *N* = 10 000 NVIDIA V100 chips, and required a total $\Sigma =3.14\times 10^{23}$ FLOPs to train to completion [17]. A single V100 has a throughput of *C* = 2.8 × 10^13^ FLOPS for half-precision floats, and so we can estimate GPT-3’s total training time in datacentre-seconds as

$$\frac{\Sigma }{C\cdot N}=\frac{3.14\times 10^{23}}{2.8\times 10^{12}\cdot 10^{4}}=1.12\times 10^{6}\;\text{s},$$

which is approximately 311 h. We know the thermal design power of a single V100 chip is 300 W and so we can safely assume a lower bound on the datacentre power usage as 3000 kW. Therefore, we estimate the total power consumed while training GPT-3 as

3000⋅311=933 000 kWh.

The emissions per kWh of the datacentre where GPT-3 was trained is 0.429 kg CO_2_e kWh^−1^ [321], leaving us with a total emission of around 400 000 kg CO_2_e.^57^

However, GPT-3 is already years old; so we will also estimate the energy used when training Google’s state-of-the-art ‘PaLM’ foundation model. Chowdhery *et al.* [270] state: ‘We trained PaLM-540B on 6144 TPU v4 chips for 1200 hours and 3072 TPU v4 chips for 336 hours including some downtime and repeated steps… [We found a] 378.5 W measured system power per TPU v4 chip…’ We can therefore calculate PaLM’s total energy usage as

378.5⋅(6144⋅1200+3072⋅336)≈3 180 000 kWh.

**Figure 31.**
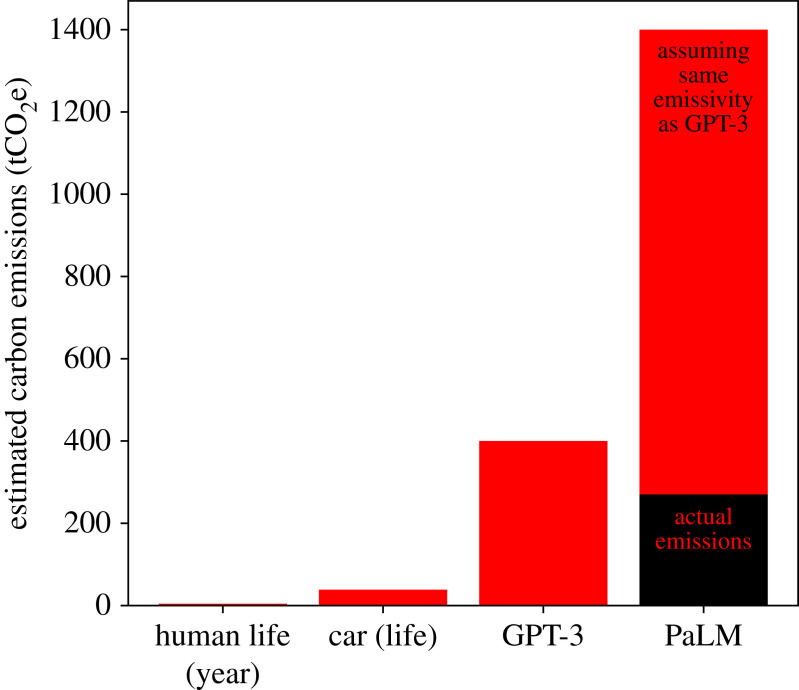
Here we contextualize the huge carbon footprints generated when training foundation models. The average person’s yearly carbon footprint is estimated as 4750 kg CO_2_e using data from Friedlingstein *et al.* [322], and the car lifetime emissions is 38 504 kg CO_2_e assuming a Mercedes-Benz C 300 d model [323].

If PaLM was trained on the same datacentre as GPT-3 (i.e. at an emissivity of 0.429 kg CO_2_e kWh^−1^), it would have emitted a staggering 1 400 000 kg CO_2_e—quadruple the average person’s lifetime carbon footprint [322] and approaching the annual emission of some small countries. Luckily, the datacentre that PaLM was trained on was far greener than that used by OpenAI, and PaLM actually produced approximately 270 000 kg CO_2_e [270], although this is still rather large. We contextualize our calculated footprints visually in figure 31.

PaLM’s contribution to figure 31 demonstrates the importance of choosing and using datacentres that run on clean energy sources when training deep learning models and make efficient use of heat output (e.g. through recovery systems). Besides this, researchers can also take care when optimizing their neural network models to reduce their carbon footprint. For instance by choosing hyperparameters through a more efficient manual or randomized search, instead of via a brute force method [324]. As stated in Strubell *et al.* [325] researchers can also combat redundant retraining of models (and thus unnecessary energy usage) by ensuring that fully trained models, data and code are released under an open licence. The publishing of a fully trained model’s energy usage, computation requirements and carbon footprint also allows downstream researchers to determine whether replication of a work is economically and environmentally viable. Calculating one’s energy usage in the spirit of openness does not have to be difficult: we have been using the excellent and user-friendly ‘Machine Learning CO_2_ Impact Calculator’ in our own work to calculate and publish the carbon footprint of our models [326]. A recommendation of this review is that an environmental impact statement should become standard practice in journal articles, conference presentations and proceedings when deep learning models (or any high-performance computing (HPC)-heavy research for that matter) is used.

## 11. Final comments, or how we learnt to stop worrying and love astronomy’s Big Data Era

To repeat our introductory statement: in every field that deep learning has infiltrated we have seen a reduction in the use of specialist knowledge, to be replaced with knowledge automatically derived from data. We have already seen this process play out in many disparate fields from computer Go [15], to protein folding [16], to natural language processing [17], to computer vision [18]. This process is already well known within the deep learning community as ‘The Bitter Lesson,’ a precept that is summarized by the quote:

The biggest lesson that can be read from 70 years of AI research is that general methods that leverage computation are ultimately the most effective, and by a large margin. [91]

**Figure 32.**
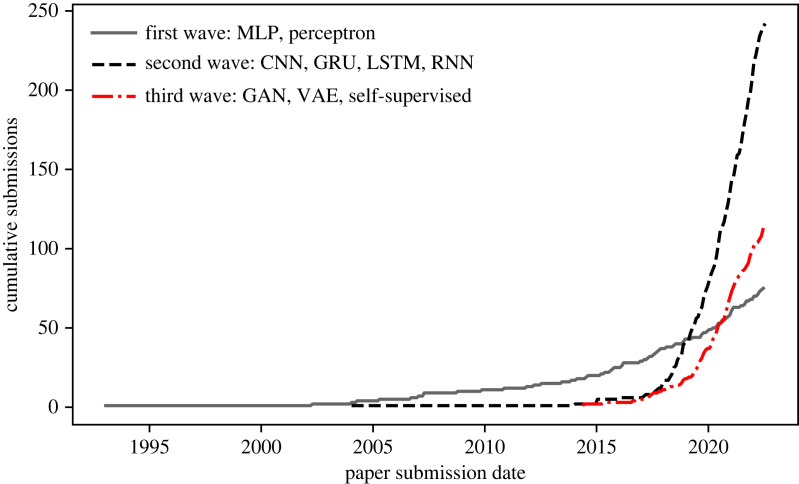
Here we see the number of arXiv:astro-ph submissions whose titles or abstracts match the terms given in the legend. We can see three distinct ‘waves’. The first corresponds to studies that use MLPs (§§2.1–3), the second corresponds to studies that use ‘deep learning’ methods that injest raw data (§§4.1–5) and the third corresponds to studies that use generative or self-supervised models (§§6–8). The raw data are in the public domain, and are available at https://www.kaggle.com/Cornell-University/arxiv.

There is no reason to believe that astronomy is fundamentally different. Indeed, within this review we have seen a narrative pointing to this conclusion (figure 32). Initial work on MLPs within astronomy required manually selected emergent properties as input (e.g. [53,75]). With the advent of CNNs and RNNs, these manually selected inputs gave way to raw data ingestion (e.g. [131,155]). Now we are seeing the removal of human supervision altogether with deep learning methods inferring labels and knowledge directly from the data (e.g. [170,177]). Ultimately, if astronomy follows in the footsteps of other applied deep learning fields, we will see the removal of expertly crafted deep learning models, to be replaced with fine-tuned versions of an all-encompassing ‘foundation’ model [173]. This process is by no means a bad thing; the removal of human bias in the astronomical discovery process allows us to find ‘unknown unknowns’ through serendipity [169,261]. Likewise, the ability to leverage data allows us to directly generate and interrogate realistic yet synthetic observations, sidestepping the need for an expensive and fragile classical simulation [13,239].

Astronomy’s relative data wealth gives us the opportunity to form a symbiotic relationship with the cutting edge of deep learning research, an increasingly data hungry field [92,280]. Many ultra-large datasets in machine learning are proprietary, and so the astronomical community has the opportunity to step in and provide a high-quality multi-modal public dataset. In turn, this dataset could be used to train an astronomical ‘foundation’ model that can be used for state-of-the-art downstream tasks (such as astronomical simulation, see §9.3.3). Finally, following recent developments in connectionism [17,273] most astronomers lack the resources to train models on the cutting edge of the field. If astronomy is to have any chance of keeping up with the Big Tech goliaths, we must follow the examples of EleutherAI and HuggingFace and pool our resources in a grassroots-style open source fashion (§9). We leave this as a challenge for the community.

## Data Availability

This article has no additional data.

## Footnotes

1 And the term is regularly misused, not only erroneously, but often cynically.

2 Deep learning referring to the use of a network constructed of many layers of artificial neurons.

3 Since its inception, AI research can be broadly categorized into two schools: ‘symbolic’ and ‘connectionist’. Symbolists see the mind as a collection of fully formed representations, and attempt to mimic human reasoning through a logical rule-based processing of these symbols. This approach contrasts with connectionist (or neural network-based) AI, which takes a bottom-up approach and simulates cognition by mimicking the way neurons in the human brain work.

4 We refer the reader to figure 1!

5 Also known by the mantra ‘cells that fire together wire together’.

6 The eagle-eyed reader may have noticed that since the derivative of the Heaviside step function is the Dirac delta function, we will only update the perceptron’s weights on an incorrect prediction. If we want to also learn from positive examples, we need to use a smoothly differentiable activation function. This is explored in the next subsection.

7 See Olazaran [32] and Metz [33] for a closer look at the conflicts and personalities that shaped AI.

8 At least, in the Western world. Connectionism continued in earnest in the Soviet Union [34,35].

9 Some controversy surrounds backpropagation’s discovery. The Finnish computer scientist Linnainmaa proposed the reverse mode of automatic differentiation and adapted the algorithm to run on computers in their 1970 (Finnish language) thesis [43]. They first published their findings in English in 1976. Werbos [41] then proposed applying an adaptation of Linnainmaa’s method to artificial neural networks. Rumelhart *et al.* [42] showed experimentally that backpropagation can generate meaningful internal representations within a neural network, and popularized the method. Here we will err on the side of caution and cite all three manuscripts. For further reading, we recommend Schmidhuber [44] and Baydin *et al.* [45].

10 If we examine equation (2.5) carefully, we can see why we add nonlinearities between the MLP layers; without activation functions equation (2.5) collapses to the equivalent of a single layer MLP!

11 Specifically, galaxies were discussed in Rappaport & Anderson [51] and point sources observed with the Infra-Red Astronomical Satellite (IRAS) were discussed in Adorf & Johnston [52].

12 At the height of the convolutional neural network architecture’s popularity in the mid-2010s, these were real problems. However, with the growth of computing power and data in recent years we are seeing a resurgence of the more general MLP model (e.g. [87–90]). This follows the prevailing trend in AI where the removal of human-crafted features and biases ultimately results in more expressive models that learn such features and biases directly from data [91,92].

13 All astronomical objects shown in the neural network diagrams within this manuscript are generated via text prompts fed into a latent diffusion neural network model [94].

14 We must note that in equation (4.1) we follow most deep learning libraries and perform a cross-correlation and *not* a convolution. However, since the weights are learnt, this does not matter; the neural network will simply learn a flipped representation of the cross-correlation.

15 As with any rule there are exceptions, such as CNNs containing a global average pooling layer [95].

16 Likewise, if φ′ is typically greater than one, the product term rapidly ‘explodes’ to infinity. This is known as the ‘exploding gradient’ problem, also first identified in Hochreiter [98].

17 ReLU is always zero if its inputs are less than 0, removing any signal for further training. This is known as the ‘dying ReLU’ problem, but is not as big of an issue as it first seems. Since contemporary deep neural networks are greatly overparametrized (see for example Frankle & Carbin [100] and other work on the ‘lottery ticket hypothesis’) backpropagation through the ReLU activation function can act as a pruning mechanism, creating sparse representations within the neural network and thus reducing training time even further [101].

18 Dropout reduces the amount of neural network overfitting—where a network performs well on the training set at the expense of performance on data it has not yet seen. One performs dropout by randomly removing a set of neurons at each training step, and using all neurons at test time. This set-up essentially trains a large ensemble of sub-models, whose average prediction outperforms that inferred by a single model.

19 Compare also the gated recurrent unit (GRU, [114]).

20 Here we loosely follow Bayer ([115], §1.3.4).

21 Which is great in theory. In practice, LSTMs still have trouble learning very long-range dependencies due to their reliance on recurrent processing [116]. Transformer networks [117] are an architecture that uses the concept of attention to address this issue. We will discuss transformer networks in §4.4.

22 In the original transformer formulation described in Vaswani *et al.* [117], the network consisted of a connected ‘encoder’ and ‘decoder’ section much like a Seq2Seq model (figure 13). Later work has found this to be an unnecessary complication. For example, the generative pretrained transformer (GPT) 2 and 3 models [17,122] consist of only decoder layers, and the bidirectional encoder representations from transformers (BERT) model consists of only encoder layers [123].

23 One can go very general with this, as DeepMind demonstrated with their ‘Gato’ transformer model [124]. Gato can predict sequences for myriad tasks, from operating a physical robotic arm, to completing natural language sentences, to playing Atari games.

24 This view demonstrates that transformers can be thought of as a class of graph neural network—a network that is tasked with learning the relationships between nodes in a graph. One can also approach this task with a feed forward neural network (§2.2; [125]), convolutional architecture (§4.1; [126,127]) or with a recurrent architecture (§4.3.3; [128]).

25 This includes most MLP applications in astronomy, see §3.

26 To avoid breaking the backpropagation chain the VAE injects noise via an external parameter, ϵ. This is described in Kingma & Welling [175] as the ‘reparametrization trick’.

27 Interestingly, this iterative approximation is similar to the approach used in the training of score-based generative models and diffusion models [179], and the similarities between the training methods of state of the art in VAE models and SBGMs are striking. For example, the Vector-Quantized VAE, Very Deep VAE and the Nouveau VAE all use a hierarchical architecture that iteratively injects latent codes that are used to produce finer and finer detail in the generated image [180–182].

28 Denoising autoencoders (§6.1) have an interesting relationship with score-based generative (or diffusion) models. As a taster, Turner [208] reframe diffusion models as a class of hierarchical denoising VAE, and Dieleman [209] show through a brief derivation that diffusion models optimize the same loss as a denoising autoencoder.

29 See appendix B in Sohl-Dickstein *et al.* [193] and appendix A in Ho *et al.* [194] for the full derivation.

30 Typically, a U-Net; see §4.3.3 for more detail.

31 A model that employs self-supervised learning is one that obtains a supervisory signal from the data itself. ‘Self-supervised learning’ as a descriptor has largely superseded the older term ‘unsupervised learning’. This is because the older term suggests that there is no supervisory signal at all—but the signal is there, just not explicitly defined by a human expert!

32 All embeddings in this subsection are normalized.

33 This self-supervised training set-up is similar to that used to train autoregressive foundation models. These models will be explored in detail in §9.1.

34 Interestingly, this process is used in the calculation for the Fréchet inception distance (FID) [223,224]. The FID acts as a measurement of the visual similarity between two datasets. The FID works by taking the penultimate layer representations from a trained Inception v. 3 model [225] for each dataset and calculating the distance between them.

35 Also compare its companion paper [226].

36 Flow-based models have not been discussed in detail in this review, but see Weng [232] for a magisterial introduction to the subject.

37 This preliminary work has been subsequently extended in Remy *et al.* [244].

38 Since the first posting of this review there have been several workshop papers presented at the 36th Conference on Neural Information Processing Systems (NeurIPS 2022) on the application of SBGMs to astronomical problems (e.g. [250–252]). Here we will highlight a particularly neat example of diffusion model application: Karchev *et al.* [251] tackle the inverse problem of strong-lensing source reconstruction and prove that a denoising diffusion restoration model (DDRM, [253]) inference scheme alongside an off-the-shelf ‘AstroDDPM’ model [13] can restore galaxies that have been through a lensing process. Remarkably, they achieved this *without any retraining or fine-tuning of the original AstroDDPM model*, demonstrating that generalist pretrained score-based models like that described in Smith *et al.* [13] can easily be repurposed for seemingly out-of-distribution downstream tasks. We will revisit the idea of pretrained models that can be repurposed for downstream tasks when we discuss ‘foundation’ models in §9.

39 See footnote 45 for commentary of this study in the context of astronomical foundation models.

40 A contrastive learning framework that unlike SimCLR does not use negative samples to learn an embedding space.

41 For now! It may be that network architecture does not matter all that much at scale, and that any sufficiently large neural network is adequate. If this is true, we will see the simplest (and most scalable) architectures win out. Although this theory has not yet been rigorously tested, we are currently seeing rumblings that suggest that this is the case (e.g. the section ‘Transformers are not special’ in [265]). Bo [266] stands as a particularly notable example of this hypothesis, showing that an attention-free RNN is capable of matching the performance of a similarly scaled transformer network. Also see footnote 12 for commentary on the performance capabilities of MLPs and transformers.

42 These models are collectively known in the literature as large language models, or LLMs.

43 For more on self-supervised learning, see §7.

44 Interestingly, the authors of Flamingo first assumed that Flamingo’s prediction of the species range of its eponymous bird was incorrect: flamingos are found in the Caribbean, South America, Africa, Europe and South Asia. However, they later realized that the picture in figure 25 is of an *American* flamingo, which is specifically found in the Caribbean and South America, so the network was right after all! See the reddit thread for the full context [271].

45 Walmsley *et al.* [274] explore in a preliminary study a ‘galaxy foundation model’ trained on Galaxy Zoo labels, and corresponding paired galaxy observations. They find that their pre-training is beneficial for training a network that performs a downstream task. However, the idea has been around for far longer than that; possibly the first demonstration of an astronomical foundation model was described 8 years earlier in Graff *et al.* [257]. Graff *et al.* [257] demonstrated that embeddings learnt with their autoencoding SkyNet network can be used for downstream tasks, but they do not use the moniker ‘foundation model’ to describe SkyNet, as the term had not yet been invented! Notably, neither study trains a model of the scale required to exhibit emergent properties or task generalizability. These ‘blessings of scale’ require data and compute at a level that has not yet been seen within astronomical connectionism.

46 Earlier work from Kaiser *et al.* [281] also demonstrated a deep learning model that could learn from disparate tasks; however, Gato is the first model that achieves this while staying within a single deep learning paradigm.

47 Of course, the reduced, useful data will be far smaller than our raw estimate here. The motivation behind this calculation is to show that even a single astronomical survey rivals the largest text dataset in size. A compilation of all useful astronomical data would certainly dwarf any contemporary text dataset, whether public or proprietary.

48 This is essentially training the model to act as a physics simulator. Viewing foundation models as world simulators is not unprecedented. This perspective has already been explored in the simulation of thousands of ‘social simulacra’ within a model online community [288], and with the simulation of participants in classic (i.e. Milgram’s shock experiment, the Ultimatum Game) and novel psychological studies [289].

49 While we revisited this subsection for our review rebuttal.

50 This is a specific example of the more general rule that ‘bazaar’ (public from conception) style open development outcompetes the ‘cathedral’ model (closed until release, or in this case closed even after release) on an equal playing field [284].

51 Although if we extrapolate from the historical trend of LLM development, OpenAI’s general research culture and direction and the time GPT-4 takes to run inference, we could arrive at the conclusion that GPT-4 is essentially a scaled-up ‘GPT-3’ model that follows a Chinchilla-optimal scaling law (§9.2).

52 Work is already being done to realize this. For example, Bowles *et al.* [302] propose a semantic natural language labelling scheme for the Galaxy Zoo evolutionary map of the universe project.

53 Naturally, no implementation is provided by Google. However, there is a fantastic MIT-licensed implementation of Imagen provided by Phil Wang and others (https://github.com/lucidrains/imagen-pytorch), and StabilityAI has a similar trained open source model released under the name ‘Stable Diffusion’ (https://github.com/Stability-AI/stablediffusion).

54 This process is also known as ‘transfer learning’.

55 Such as Green Bean Galaxies [313], or SETI events akin to the ‘Wow!’ signal [314].

56 We would compare GPT-4, but OpenAI has neglected to disclose any information regarding the training routine of the network in their ‘Technical Report’ [26].

57 We must keep in mind that this estimate is a lower limit. We do not include CPU power, cooling or any other overheads in our calculation, never mind the cost to do a full hyperparameter sweep!
